# Fundamentals of Flexoelectricity, Materials and Emerging Opportunities Toward Strain‐Driven Nanocatalysts

**DOI:** 10.1002/smll.202406726

**Published:** 2024-11-06

**Authors:** Mieszko Kołodziej, Niwesh Ojha, Michał Budziałowski, Karol Załęski, Ignasi Fina, Yogendra Kumar Mishra, Kamal Kishore Pant, Emerson Coy

**Affiliations:** ^1^ NanoBioMedical Centre Adam Mickiewicz University Wszechnicy Piastowskiej 3 Poznan 61‐614 Poland; ^2^ Department of Chemical Engineering Indian Institute of Technology Roorkee Roorkee Uttarakhand 247667 India; ^3^ Institut de Ciència de Materials de Barcelona (ICMAB‐CSIC) Campus UAB Bellaterra Barcelona 08193 Spain; ^4^ Smart Materials, NanoSYD, Mads Clausen Institute University of Southern Denmark Alsion 2 Sønderborg 6400 Denmark; ^5^ Center for Sustainable Energy Indian Institute of Technology Roorkee Roorkee 247667 India; ^6^ Catalytic Reaction Engineering Lab, Department of Chemical Engineering Indian Institute of Technology Delhi New Delhi 110016 India; ^7^ University of Saskatchewan Saskatoon SK S7N 5A2 Canada

**Keywords:** energy conversion, external stimulus, flexoelectricity, nanocatalysts, photocatalysis

## Abstract

Flexoelectricity, an intrinsic property observed in materials under nonuniform deformation, entails a coupling between polarization and strain gradients. Recent catalyst advancements have reignited interest in flexoelectricity, particularly at the nanoscale, where pronounced strain gradients promote robust flexoelectric effects. This paper comprehensively examines flexoelectricity, encompassing methodologies for precise measurement, elucidating its distinctions from related phenomena, and exploring its potential applications in augmenting catalytic properties. So far, the greatest potentials are based on lead strontium titanate (PST) and other metallic titanates such as titania (TiO_2_), strontium titanate (STO), barium strontium titanate (BST) sulfates (MoS_2_, ZnS) and halide perovskites (with archetype XPbI_3_). This review explores the promise of flexoelectric properties in addressing material and photocatalytic challenges, such as charge carrier recombination and ineffective surface charge separation. Additionally, it sheds light on the synergy with emerging paradigms like photo‐flexo catalysis and synergistic flexo‐piezo catalysis, specifically focusing on selective chemical transformations like green hydrogen production. Current limitations related to the usage of photoflexoelectricity for photocatalysis are mostly the stability of the used substance (susceptibility to photodegradation) or the voltage values, which represent the inferior potential for specific practical applications. This work underscores the indispensable role of flexoelectricity in catalysis and its capacity to steer future research and technological advancement.

## Graphical abstract

This review examines flexoelectricity, a property where bending materials create electric charges. It explores how this effect can improve catalysts, especially at the nanoscale. The paper discusses measuring flexoelectricity and its advantages in catalysis, like reducing wasted energy. The review also explores combining flexoelectricity with new technologies for cleaner energy production. While limitations exist, flexoelectricity has exciting potential for future advancements in catalysis.

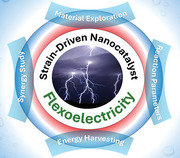

## 1 Introduction

Hydrogen is an abundant element on Earth. It has a relatively simple chemical dissociation from water and highly energetic recombination with oxygen, making it a promising energy source for our society's current and future needs. Unlike fossil fuels and coal, which are nonrenewable and harmful to the environment, hydrogen can be generated through water splitting integrated with renewable energy sources.^[^ ^1^ ^]^ This process can be achieved using catalytic methods, such as electrolysis, photocatalysis, and photoelectrolysis. Catalysts play a significant role in the global economy, accounting for over 35% of the global gross domestic product, and are used to produce nearly 80% of all manufactured goods.^[^ ^2^ ^]^ As a result, it is important in the modern chemical industry to concentrate efforts on understanding the catalyst surface mechanism to increase the reaction conversion rates or product. Over the last decade, significant research efforts have been made to improve the key properties of the catalytic active sites and how to tweak them to create a specific reaction with a higher yield. Altering the crystallinity, phase, exposed facet, and morphology has emerged as an important strategy to achieve this goal.^[^ ^3^, ^4^ ^]^ The purpose of studying the interfacial charge transfer in chemical reactions is to identify the driving force behind the reaction, whether it be heat, light, or electricity.

Alternative efficient sources capable of driving the water splitting like external mechanical or acoustic stimuli (e.g., vibrations) have not been widely investigated on catalysis. Among possible candidates for harvesting such energy and transforming it in available electron/hole pairs to drive catalytic reactions are piezoelectric,^[^ ^5^ ^]^ triboelectric,^[^ ^6^ ^]^ and ferroelectric^[^ ^7^ ^]^ materials, each with an interesting applicability and performance. Nevertheless, a not very well‐known effect, flexoelectricity, has entered the field, and its potential for generating high electrical outputs and efficient catalysis can tremendously influence the field of energy conversion.

This review will discuss the possible and current catalytic applications that are influenced by the flexoelectric. We will also profit from current developments in mechanically driven catalysis, an emerging field that aims to enhance catalysis without needing light or thermal energy input.^[^ ^8^ ^]^ Mechanical triggering is the mechanism by which a mechanochemical activation is induced, promoting the catalytic process.^[^ ^9^ ^]^ The significance of such characteristics extends beyond merely maximizing product yield under existing conditions. They also prove to be valuable when integrating these chemical processes with renewable energy sources. Consider, for instance, the role of interfacial charge across the electrodes as the reaction mixture flows in kilowatt‐scale electrolyzes for the electrocatalytic chemical transformation of clean fuel production. With regards to solar energy, the invention of solar cells based on silicon ≈70 years ago marked a significant milestone. This development involved the separation of carriers by a p–n junction, generating an electrical field and opening up new avenues for research.^[^ ^10^ ^]^ However, they are still a mainstay of the solar industry. Water splitting driven by solar cells using photocatalysis is promising for cost‐effective hydrogen production.^[^ ^11^, ^12^ ^]^ However, there is still a considerable difference between the theoretical efficiency and the experimentally obtained values for specific materials. For instance, the theoretically estimated efficiency limit for a tantalum nitride is about 17%, whereas the experimental results showed an efficiency of <1%.^[^ ^13^ ^]^ Polman et al. reviewed 16 different photovoltaic materials and reported efficiencies of about 10–29%.^[^ ^14^ ^]^ It was discovered later that the flexoelectric effect can boost the efficiency of the photovoltaic effect. ^[^ ^15^ ^]^ Even so, these results pose several questions, such as short‐circuit current increasing by an applied force, not mentioning contact between electrodes and the sample, and the large flexo photovoltaic current (in the scale of ∼pA), which is close to the bottom line of the current industrial standards. Similar results have been reported for centrosymmetric BiVO_4_, where different polishing methods tuned the photovoltaic properties; thus, the role of different stresses on the photovoltaic effect was noticed.^[^ ^16^ ^]^ Applying the strain gradients to enhance the photovoltaic effect has been already examined for over a decade.^[^ ^17^, ^18^ ^]^ To date, several new reports demonstrating the enhancement of photovoltaic current directly by the flexoelectric effect have been published, providing a few examples: Fe‐doped LiNbO_3_ bulk single crystals,^[^ ^18^ ^]^ (K, Na)NbO_3_ / vinylidene fluoride‐trifluoroethane nanocomposites,^[^ ^19^ ^]^ two‐dimensional MoS_2_,^[^ ^20^ ^]^ and (1 − *x*)(Bi_0.5_Na_0.5_)TiO_3_ – (*x*)BiFeO_3_ ceramics.^[^ ^21^ ^]^ Nevertheless, the polarization caused by the flexoelectric effect was not directly evaluated, leading to many open questions and partially a controversy on this topic.^[^ ^22^ ^]^ The influence of the flexoelectric effect on the photovoltaic effect was recently reviewed and marked that further studies of the flexoelectric and flexophotovoltaic effects are still necessary for possible future progress.^[^ ^23^ ^]^ Considering all of the above consequences, this is the ideal moment in the field to summarize and review the technological development for newcomers, outlining the challenges and opportunities in this fascinating topic for the next 10 years and the future. This review will first introduce the basics of flexoelectricity and its detection methods. We will highlight the most interesting materials: Strontium titanate (STO), barium titanate (BTO), barium–strontium titanate (BST), strontium ruthenate (SrRuO_3_) titanium and zinc oxide (TiO_2_ and ZnO), halide perovskites and at the end, dichalcogenides. Finally, we summarize the most important and latest developments and the open prospects for further research in flexoelectric and photoflexoelectrics.

### 1.1 What is Flexoelectricity?

Flexoelectricity in solids, also known as the flexoelectric effect, is a mechanism that enables the presence of electric polarization owing to a strain gradient (direct flexoelectric effect) or the existence of a strain owing to an electric field gradient (converse flexoelectric effect). Flexoelectric phenomenon was observed for the first time in 1968 by Bursian and Zaikovskii in ferroelectric films,^[^ ^24^ ^]^ and since then, it has been well understood and explained. The advantage of the flexoelectricity phenomenon over the piezoelectric effect is that it does not need to meet any structural symmetry requirements (unlike piezoelectricity, ferroelectricity, or ferroelasticity). Therefore, it is present in any crystalline insulating material. The flexoelectric response of a material is directly proportional to its flexoelectric coefficient and the strain gradient. Although ceramics are fragile, their flexoelectric coefficient is relatively high (≈10^−6^ C m^−1^).^[^ ^25^ ^]^ On the other hand, polymers are known for their capability to generate much higher strain gradients without breaking. However, they exhibit much lower flexoelectric coefficients (≈10^−8^ to 10^−9^ C m^−1^).^[^ ^26^ ^]^ This illustrates the interdependence on mechanical stiffness, strain and polarization in this effect.

Despite the low polarization of the flexoelectric effect, due to the bulk/surface ratio, it conversely becomes more dominant at the nanoscale. It is also worth mentioning that the flexoelectric coefficients obtained experimentally (e.g., (Ba, Sr)TiO_3_)^[^ ^27^ ^]^ are much higher than those calculated theoretically, which suggests that, even if the flexoelectric effect is size‐dependent and much higher in smaller devices, and it could have a much larger contribution at the macroscale than expected. Furthermore, the flexoelectric effect is also known to be susceptible to structural defects such as grain boundaries,^[^ ^28^ ^]^ doping, and dislocations,^[^ ^29^ ^]^ which is also the case for experimental value differences in single crystal measurements^[^ ^30^ ^]^ and their related theoretical calculations.^[^ ^31^ ^]^ Currently, flexoelectricity is being studied for many applications, such as in biological systems,^[^ ^32^ ^]^ energy harvesting,^[^ ^33^ ^]^ triboelectricity,^[^ ^34^ ^]^ water splitting or purification,^[^ ^35^, ^36^ ^]^ actuators,^[^ ^37^ ^]^ polarization switching,^[^ ^38^ ^]^ domain tailoring,^[^ ^39^ ^]^ and sensors.^[^ ^40^ ^]^ For instance, nanogenerators based on the piezoelectric effect were proposed in 2006,^[^ ^41^ ^]^ and are nowadays enhanced by the flexoelectric effect.^[^ ^42^ ^]^

### 1.2 Disclosing Flexoelectricity from Piezoelectric, Ferroelectric, and Ferroelastic Effects

Crystals with either symmetric or asymmetric atomic arrangements can generate electric polarization when subjected to external mechanical forces. In symmetric structures with inversion centers, where the arrangement of atoms is mirrored across specific points, the overall net movement of electric dipoles within the crystal cancels out. In contrast, crystals with asymmetric structures lacking inversion centers can exhibit noncentrosymmetric crystal structures and possess *piezoelectric* properties, e.g., AlN. These materials present polarization (or bound charges) on opposite surfaces under mechanical stress (bending, compression, and stretching). In addition, few piezoelectric materials belong to the group of ferroelectric materials, meaning that they have two states of polarization that can be changed by an external electric field. In this regard, PbTiO_3_(PTO), Pb*_x_* Zr_1–_ *_x_* TiO_3_(PZT), BaTiO_3_ (BTO), and BiFeO_3_ (BFO) are well‐known ferroelectric materials with a high piezoelectric response. Piezoelectric and ferroelectric materials have wide applications in many fields, typically depending on the need for the remanence of states (i.e., memories), or current/event‐triggered effects (i.e., mechanical actuators or sensors).

In addition, the ferroelectric materials exist in different stable phases owing to spontaneous lattice reorientations in the presence of external mechanical energy, eventually serving as a reversible switch between different crystal phases. These properties are commonly known as ferroelasticity. For example, nitinol (nickel–titanium) is a standard ferroelastic alloy with either super‐elasticity or shape memory, depending on the nickel‐to‐titanium ratio at room temperature.^[^ ^43^ ^]^

**Figure 1:**
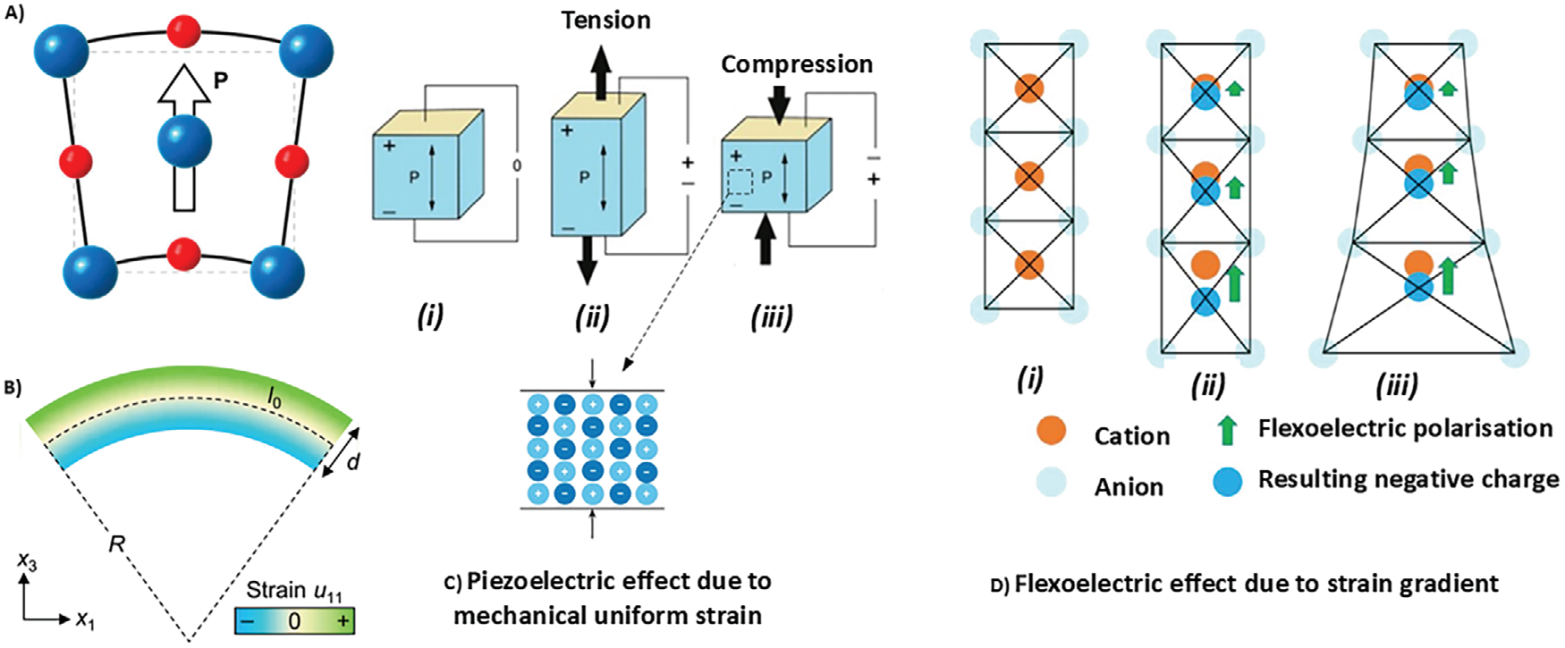
A) Change in centrosymmetry under the influence of B) strain gradient causes flexoelectricity generation; C) strain gradient can shift the center of positively charged ions from that of negatively charged ions, resulting in a nonzero electric dipole moment. D) A strain gradient ∂u/∂x can induce electric polarization P through flexoelectricity when a material is bent. *R*, *d*, and *I*_0_ indicate the radius of curvature, thickness, and original lateral length of the material being bent, respectively. Image adapted with permission from references.^[^ ^46^, ^57^ ^]^ Copyright 2023, Springer Nature; Copyright 2020, AIP Publishing.

Usually, all the centrosymmetric and nonsymmetric materials under the influence of an external force (e.g., bending deformation) cause inhomogeneous strain gradient creation along the bending direction, separating the positive and negative charge centers and forming a polarization electric field.^[^ ^36^ ^]^ Eventually, it generates a net polarization inside a crystal due to the non‐uniform distribution of ions under a strain gradient, generally caused by splay or bent deformations, disrupting the inversion symmetry.^[^ ^44^ ^]^ As a result, flexoelectric effects are generated. Similarly, the converse flexoelectric effect is formed when coupling the electric field gradient and mechanical stress.^[^ ^45^ ^]^ Flexoelectricity and piezoelectricity are two related but distinct phenomena that involve generating electrical charges in response to mechanical deformations, where the first is generated owing to a strain gradient, and the second is generated by uniform strain (**Figure** **1**C,D).^[^ ^46^ ^]^ What makes a huge difference is that the piezoelectric effect is strictly limited by the crystal symmetry (noncentrosymmetric), which is not the case for flexoelectricity (it is present in any crystalline material). More interestingly, the flexoelectric effect does not fade away at a specific temperature, which means that, theoretically, the devices based on this effect might operate from liquid helium temperatures up to the melting point of a given material.^[^ ^47^ ^]^ Nonetheless, in catalytic applications, those materials exhibiting piezoelectric response have been actively explored for catalytic conversion, mainly in water splitting, wastewater purification, dye degradation, and others, owing to the scale of the effect.^[^ ^48^, ^49^, ^50^ ^]^ Additionally, ferroelectric materials and their effective charge coupling between ferroelectric polarization and other materials under the illumination of light or ultrasonication have also been studied.^[^ ^35^, ^51^, ^52^ ^]^ However, the role of flexoelectric materials in catalytic applications is still unclear and has yet to be explored in depth. This study focuses on the potential uses of flexoelectricity in catalytic applications, highlights vital characteristics, and briefly describes various flexoelectric materials. We also discuss the diverse range of chemical reactions that can be carried out under the combined effect of flexoelectricity and photocatalysis, commonly known as the photo‐enhanced flexoelectric effect.

### 1.3 Typical Flexoelectricity Characteristics: Symmetry, Dependency, and Strength

**Figure 2:**
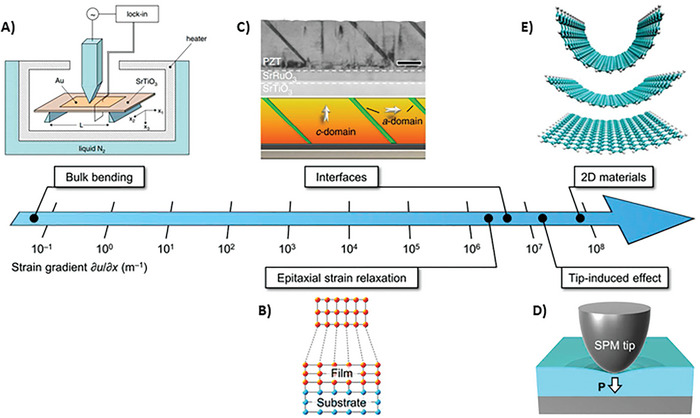
Possible range of strain gradients, achievable via A) bulk bending, B) epitaxial strain relaxation, C) interfaces, D) tip‐induced effects, and e) low‐dimensional systems. Panel (A) illustrates the experimental setup for measuring flexoelectricity in bulk SrTiO_3_ single crystals. The image taken with permission from reference.^[^ ^58^ ^]^ Copyright 2007, American Physical Society. Panel (B) shows a schematic of the strain relaxation in the epitaxial thin films. The image is reproduced with permission from reference.^[^ ^59^ ^]^ Copyright 2011, American Physical Society. Panel (C) shows a transmission electron microscopy image of the ferroelastic *a*‐ and *c*‐domains in the epitaxial Pb(Zr,Ti)O_3_ thin films. Image taken with permission from reference.^[^ ^60^ ^]^ Copyright, 2013, Springer Nature. Panel (D) schematically depicts the flexoelectric polarization generated by the scanning probe microscope (SPM) tip pressing the surface of the material. Image taken with permission from reference.^[^ ^61^ ^]^ Copyright 2020, Springer Nature. Panel (E) shows a schematic of the flexed graphite membranes. Image taken with permission from reference.^[^ ^62^ ^]^ Copyright 2008, American Physical Society.

As mentioned above, the flexoelectric effect is not constrained by any crystalline or structural requirements; therefore, it is always present in crystalline materials. The modeling of the flexoelectricity in solids started back in 1964^[^ ^53^ ^]^ and later in 1968,^[^ ^54^ ^]^ followed by theoretical modeling and extension of microscopic theories,^[^ ^55^, ^56^ ^]^ which leads to the conclusion that a flexoelectric response is always generated when the crystalline material is deformed (Figure 1A,B). Mathematically, the flexoelectric effect is a second‐order effect, which is directly proportional to the product of the strain gradient and electric field gradient and can be written as follows (Equation 1):

(1) $$P_{i}=\mu_{\mathrm{ijkl}}\frac{\partial \varepsilon_{\mathrm{jk}}}{\partial x_{l}}$$

where *P_i_* stands for the flexoelectric polarisation, *µ_ijkl_* is the flexoelectric coefficient, *ɛ_jk_* is the elastic strain and *x_l_* is the coordinate. The flexoelectric coefficient is directly proportional to the dielectric susceptibility and exhibits a linear dependence, which can be expressed as (Equation 2):

(2) $$\mu_{\mathrm{ijkl}}=\chi_{\mathrm{ij}}\gamma_{\mathrm{kl}}\left(\frac{e}{a}\right)$$

where *χ_ij_* is the dielectric susceptibility, γ*_kl_* is a tensor of the material parameter (constant), *e* is the electron charge, and *a* is the lattice constant.^[^ ^55^ ^]^ Flexoelectricity is related to the size of the material. Although it is almost negligible in bulk‐size materials, it is dominant at the nanoscale. The dependence of the strain gradient on size is shown in **Figure** **2**. Additionally, it shows the possibility of designing unique strain‐polarization structures that can generate strain gradients when external pressure is applied. Additionally, the result would be a high electromechanical signal, which could be similar to piezoelectric‐based systems but without the piezoelectric effect.

In thin films, the flexoelectric effect can be used to switch the polarization.^[^ ^63^ ^]^ The lattice mismatch between the piezoelectric film and substrate can also lead to the enhancement of a strain gradient originating from the lattice mismatch between the film and substrate materials epitaxial films, relaxation along the thickness can occur via different mechanisms, which leads to a strain gradient in these structures. The magnitude and direction of flexoelectric polarization generation depend strictly on the direction of the strain gradient. In addition, flexoelectricity can be affected by the crystal structure, chemical composition, and the magnitude and direction of the applied strain gradient. However, the flexoelectric effect (nC m^−1^) is generally lower than the piezoelectric effect (pC N^−1^).^[^ ^64^, ^65^ ^]^ As a simple comparison of these coefficients, it was proposed that a piezoelectric coefficient of 1 pC N^−1^ could be similar to a flexoelectric coefficient of ≈1000 nC m^−1^.^[^ ^66^ ^]^ This is, perhaps, the main reason why the flexoelectric effect, when discovered, did not attract significant interest from researchers. According to other electromechanical effects, these values represent inferior potential for practical applications.

Given these numbers, typical modeled flexoelectric coefficients (scale of 10^−9^ to 10^−8^ C m^−2^ that can induce polarization ≈10 times lower (Equation 1), when considered, their values are relatively small when compared to those of known ferroelectric materials (where polarization is in the scale of 10^−2^ C m^−2^.^[^ ^67^, ^68^ ^]^ This leads to the conclusion that a material system with a much more significant strain gradient is necessary for future progress and the possibility of applying flexoelectric materials.^[^ ^57^ ^]^ However, this changes significantly in ferroelectric materials with very high dielectric permittivity and flexoelectric response, known as the giant flexoelectric effect.^[^ ^57^, ^69^, ^70^ ^]^ The presence of the giant flexoelectric effect caused the flexoelectric topic to return alive, and a wider group of materials are being actively investigated.^[^ ^57^, ^70^, ^71^, ^72^ ^]^

## 2 Flexoelectric Materials

In this section, we will describe some of the most studied materials in the field of flexoelectricity and generally describe their properties and some of their particular applications.

### 2.1 Strontium Titanate (STO)

STO is a well‐known flexoelectric material with the chemical formula SrTiO_3_. At room temperature, it is characterized by a perovskite‐type centrosymmetric structure (*Pm* $\bar{3}$ *m*) with a lattice parameter of 3.905 Å.^[^ ^73^ ^]^ It has a density of 5.18 g (cm^3^)^−1^ and has a very high dielectric constant of 300. In addition, it is known for its structural flexibility, high stability, and nontoxicity.^[^ ^74^ ^]^ The flexoelectric coefficient measured in STO was estimated to be 1–10 *×* 10^−9^ C m^−1[^ ^30^, ^75^ ^]^ and is comparable with standard dielectrics. STO is typically selected to study the flexoelectric response because the induced polarization is only caused by a nonuniform mechanical strain gradient.^[^ ^30^, ^45^ ^]^ It also allows for determining all tensor components using a single crystal of different orientations.

Various additional effects can enhance the electric polarization in STO. For example, piezoelectricity may still occur at the STO surface, where the centrosymmetric structure breaks.^[^ ^73^ ^]^ Polarization may also occur due to noncrystal phases, inner strain presence, and the creation of polar nanoregions.^[^ ^75^ ^]^ Finally, the polarization is highly temperature‐dependent. When cooled, STO undergoes a cubic to tetragonal phase transition at 105 K and acquires a ferroelastic character. Cooling further at 50 K also exhibited a high piezoelectric response.^[^ ^76^ ^]^

STO is a fascinating material with many applications in various fields, such as material science and rapidly growing micro‐ and nanotechnology. They are applied in micro‐ and nano‐electromechanical systems (MEMS/NEMS),^[^ ^77^ ^]^ optoelectronic devices,^[^ ^78^, ^79^ ^]^ capacitors,^[^ ^80^, ^81^ ^]^ and dynamic random‐access memory (DRAM).^[^ ^82^ ^]^ Other application fields include charge transportation,^[^ ^83^ ^]^ defect formation,^[^ ^84^ ^]^ domain tailoring,^[^ ^85^ ^]^ and flexoelectric effect.^[^ ^86^, ^87^, ^88^ ^]^ Different methods usually use STO as a substrate for epitaxial growth of oxide thin films.^[^ ^89^, ^90^ ^]^ It is an excellent electrical insulator^[^ ^91^ ^]^ or conductor that doping can easily tune.^[^ ^92^ ^]^ Doped STO can also be used as a high‐temperature superconductor.^[^ ^93^, ^94^ ^]^ STO can also be used in thermoelectric applications. It can be used to convert waste heat into electricity, for example, in thermoelectric generators and coolers.^[^ ^95^ ^]^ It can also be applied in various photonic devices, such as optical coatings^[^ ^93^ ^]^ and waveguides,^[^ ^94^ ^]^ owing to its relatively high refractive index and transparency in the visible and near‐infrared spectra.^[^ ^96^, ^97^ ^]^

More interestingly, STO is also known as an active UV light photocatalyst, with significant applications in several processes.^[^ ^98^ ^]^ It acts as an n‐type semiconductor characterized by a wide bandgap of 3.22 eV, similar to TiO_2_ (commonly used in different photocatalytic applications).^[^ ^99^ ^]^ STO has been explored for scalable water splitting for solar‐to‐hydrogen energy conversion.^[^ ^100^ ^]^ STO also has a lot of potential for photocatalysis for water splitting and pollutant degradation.^[^ ^101^ ^]^ This is where single‐crystalline STO has been assessed as a photoanode. Therefore, owing to its flexoelectric properties described here, nano/micrometric crystalline STO can be incorporated, alone or with other piezoelectric composite materials, to enhance the generated polarization by improving surface sensitivity as a catalyst.^[^ ^87^ ^]^

### 2.2 Barium Titanate (BTO)

BaTiO_3_ (BTO) is a well‐known piezoelectric material. In natural form, it has a perovskite structure consisting of TiO_6_ octahedra and BaO_12_ octahedral,^[^ ^102^ ^]^ which, with changing temperature, undergo three phase transitions. Starting at cubic (*Pm* $\bar{3}$ *m*) structure above 393 K, it transverses into a tetragonal (*P4mm*) when cooled below. Next, at 278 K, the structure changed to orthorhombic (*Amm2*); below 183 K, it became rhombohedral (*R3m*). These structural transitions, especially near room temperature, make BTO interesting from an industrial point of view.^[^ ^103^ ^]^ BTO is also known as a noncentrosymmetric wide‐bandgap material that exhibits the bulk photovoltaic effect.^[^ ^104^ ^]^ BTO is considered a potentially applicable material for flexoelectricity and flexophotocatalysis.^[^ ^105^ ^]^ Single crystals and polycrystalline materials of BTO have been investigated from piezoelectric and ferroelectric points of view for decades.^[^ ^106^ ^]^ As previously mentioned, they are both well‐studied piezoelectric materials. Additionally, BTO is known to be the first polycrystalline material in which ferroelectricity was observed.^[^ ^103^ ^]^ More importantly, it also exhibits a notable flexoelectric coefficient.^[^ ^107^ ^]^ Navares et al. characterized a single crystal of BTO enriched with oxygen vacancies, which was initially expected to decrease its electrical resistance. When vacancies are present, it becomes an n‐type semiconductor with no charge on its surface. The results of the flexoelectric tests showed that the semiconducting state was hundreds of times larger than the re‐oxidized BTO. Subsequently, these crystals were annealed in an oxygen atmosphere and retained their high permittivity (vacancies healed). In conclusion, the oxygen vacancies induced flexoelectricity in the sample. Moreover, this process can be reversed through oxidation. Its flexoelectric coefficient was estimated to be 1 *×* 10^−6^ C m^−1^.^[^ ^108^ ^]^ Furthermore, in a similar study published by Dai et al., single crystalline BTO (enriched with oxygen vacancies) films on graphene/germanium were prepared. It formed a textured film following the Volmer–Weber growth mode^[^ ^109^ ^]^ with enhanced flexoelectric properties, which confirmed that oxygen vacancies are desirable for enhancing the flexoelectricity of BTO.^[^ ^110^ ^]^

Flexible BTO nanofibrous membranes were fabricated to enhance the efficiency of the piezocatalysis. When catalytic processes were additionally supported by ultrasonic vibrations, they achieved a degradation degree of 96% in 60 minutes, with superb reusability and no time ‐consuming processing requirement.^[^ ^111^ ^]^ Recently, the layered barium dititanate (BaTi_2_O_5_), characterized by centrosymmetric trigonal crystal structure in nanopowder form was synthesized, and their catalytic performance in hydrogen production and organic pollution degradation was tested. The enormously high efficiency of this flexocatalytic material was confirmed in both hydrogen production and degradation. The authors rechecked the performance for 3 days, in 3‐h cycles. The production of hydrogen was rising every hour, and each day, it was higher, reaching a value of 1156.3 µmol g^−1^ h^−1^ on day 3. A degradation degree of 96% in 45 min was reported. The stability after the catalytic process was additionally confirmed, with only a minor change on the catalysts’ surface.^[^ ^112^ ^]^

### 2.3 Barium Strontinum Titanate (BST)

(Ba_1–_ *_x_* Sr*_x_*)TiO_3_ (BST) is a ceramic where *x* ranges from 0 to 0.8. Essentially similar to BTO, but with increasing Sr content, the polarization becomes complicated by antiferrodistorsive and ferroelectric instabilities.^[^ ^113^ ^]^ Detailed studies and the phase diagram of BST show that the behavior of this system is similar to that of other similar systems (such as KTaO_3_ and STO) that are based on ferroelectrics in their initial stage. The replacement of Ba in STO initially results in the formation of a glass‐like state and, later, a ferroelectric phase. When the Sr/Ba ratio crosses 1/5, the transformation of *the m3m* to *the 4mm* phase occurs.^[^ ^114^ ^]^ Generally, the flexoelectric coefficient is larger for higher Ba and lower Sr contents in BST and lies at a value of 100 *×* 10^−6^ C m^−1^. These values make BST a promising material for future development.^[^ ^115^ ^]^ In 2014, the BST/Ni_0.8_Zn_0.2_Fe_2_O_4_ composite had an even higher flexoelectric coefficient, which was in the range of 128 *×* 10^−6^ C m^−1^.^[^ ^116^ ^]^ BST was also a material used in a combination of soft and hard flexoelectrics. Ceramic (Ba_0.67_Sr_0.33_)TiO_3_ nanofiber and polyvinylidene fluoride (PVDF) as a thin film was fabricated, leading to a flexoelectric response ≈3–4 times higher when compared to pure PVDF^[^ ^117^ ^]^ (notwithstanding that the value was still in the range of ≈10^−8^ to 10^−9^ C m^−1^, which is relatively small). Still, recent studies have demonstrated that the combination of certain materials can e the flexoelectric effect.

The BST nanorods were recently tested for pollution degradation and hydrogen production efficiency. Poled piezo‐photocatalysis shows hydrogen production efficiency of about 411.5 µmol g^−1^ h^−1^ in 120 min, almost twice the results reported this year for BTO (249.5 µmol g^−1^ h^−1^ on a day 1). The degradation rate constant *k* reached a value of 0.0447 min^−1^_._ The absorption spectra suggest a degradation degree of 96% after about 60 min.^[^ ^118^ ^]^

### 2.4 Metamaterials, Topologies and Vacancy‐Induced Flexoelectricty

For the two‐dimensional topology optimization of flexoelectric metamaterials with apparent piezoelectricity, Greco et al. studied the theoretical and computational framework.^[^ ^119^ ^]^ They compared metamaterials to flexoelectric nanostructures and found that multiscale structures allow them to transcend at the nanoscale, where flexoelectricity is important, and make this effect available for electromechanical transduction at meso‐ or macroscales. Various capabilities of the actuator and sensor have been optimized, resulting in the numerical approximations of the Pareto fronts concerning such piezoelectric responses and area minimization. Nevertheless, the identified designs are significantly diverse, highly anisotropic, and more complex based on the goal functionality, stress/strain sensors, and actuators. These findings reveal a nontrivial structure–property link in piezoelectric flexoelectric metamaterials.

Similarly, Olson and Mark observed that the contacting shapes, or asperities (size and shape), are influenced by information about the material, geometry, gradient elasticity, and electronic transport. They further validated the qualitative agreement with experimental findings.^[^ ^120^ ^]^ Thus, scaling rules were provided that can be exploited to design flexoelectric‐based devices better. Their study and research also suggest a strong correlation between flexoelectricity and triboelectricity, which can have significant industrial implications.

**Figure 3:**
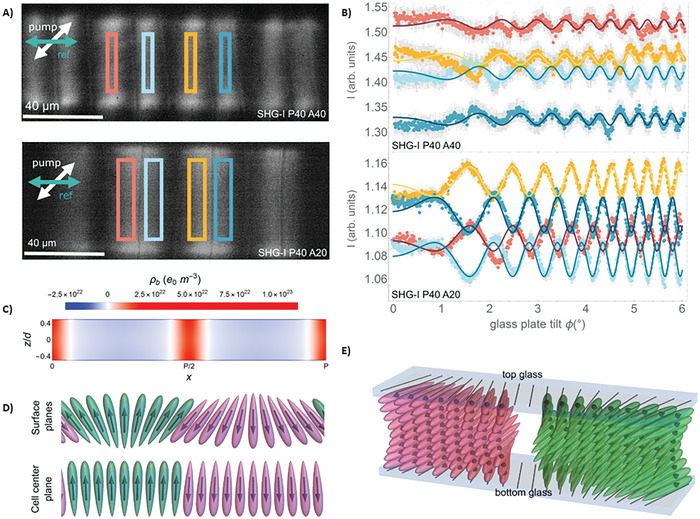
Polarization patterning proved by SHG interferometry in periodic splay photopatterned structures. A) SHG interferometry images of periodic‐splay structures Splay‐P40A40 and Splay‐P40A20, with periods of 40 µm and maximum splay angles of 40° and 20°, respectively (splay curvatures of 0.1 and 0.05 µm^−1^). The incoming polarization of the pump laser was at 45° as indicated by the white arrow, the reference signal was horizontal, and the signal was collected after a horizontal analyzer. B) SHG interferograms correspond to the highlighted areas with matching colors, C) Calculated charge distribution –∇ P across the cell thickness *d*. Schematic representation of the surface D) with cell center planes and 3D sketch E) showing the flexoelectric coupling between the splay deformation and the polarization direction. Image adapted with permission from reference.^[^ ^121^ ^]^ Copyright 2023, Springer Nature.

Furthermore, Sebastián et al. created splay structures that geometrically defined the polarization direction using the flexoelectric effect.^[^ ^121^ ^]^ They developed periodic polarization structures and showcased the potential for polarization guidance by embedding splay structures in uniform backgrounds. Eventually, this opens a promising new path for the design of ferroelectric nematic‐based photonic structures with unique polarization‐patterning capabilities. Their observations using second harmonic generation microscopy (SHG‐M) and polarizing optical microscopy (POM) could not distinguish between structures whose sign alternates with the splay direction. By altering the phase of a reference SHG signal, regions with opposite polarity could be identified because the generated SHG light phase depends on the polarization direction and the breaking of centrosymmetry. For reference, in a liquid–crystal compound having a 1,3‐dioxane unit in the mesogenic core (DIO) material in a beta barium borate (BBO) crystal, DIO was positioned such that the periodic stripes and the SHG polarization were perpendicular to each other.^[^ ^122^ ^]^ Contiguous regions with opposite splay and divided by disclination lines have opposite directions of *P*, as shown by the SHG‐I measurements (**Figure** **3**A,B), because the generated signal has a 180° phase shift between them. Their findings provide an experimental demonstration of how to design polarization structures by taking advantage of the flexoelectric coupling between polarization and orientational deformation. Even in nonpolar liquid crystals (NLCs), splay and bend director distortions in liquid crystals (LCs) can lead to an electrically polarized medium. As a result, these structures shift toward the center of the splay in response to any asymmetry in the splay structure, which carries the opposite charge (Figure 3C).

The photopatterned splay structure exhibits structural relaxation upon additional cooling, as evidenced by the emergence of domain walls that begin at the structure's edges and progress toward the center. Move in a disclination‐line‐oriented manner. With a minor distortion surrounding the recently formed domain walls, the optical transmission texture in a liquid–crystal compound having a 1,3‐dioxane unit in the mesogenic core (DIO) material remains unchanged and consistent with the observations of the dragonfly‐shaped distortions. The surface (Figure 3D) is schematically represented with cell center planes, and the 3D sketch (Figure 3E) illustrates the flexoelectric coupling between the polarization direction and the splay deformation. Furthermore, Ji et al. used the Von Kármán strain theory and extended linear theory to analyze the flexoelectric effect^[^ ^123^ ^]^ in the considerable deflection bending of a circular piezoelectric microactuator. The corresponding governing equations and boundary conditions have been obtained using the variational principle and the differential quadrature approach. The resulting substantial deflection in supported plates and clamped plates is used to explore the coupling between the flexoelectric and piezoelectric effects. The flexoelectric effect reduces the electromechanical coupling response of the piezoelectric layer. In this case, when the piezoelectric layer is turned over, the bending deformation caused by the piezoelectric effect reverses, thereby increasing the electromechanical coupling response. Moreover, the size‐dependence flexoelectric effect made a larger contribution when the actuator thickness was less than 1 µm, and as the thickness increased, the flexoelectric effect became less noticeable until the actuator was 20 µm thick or thicker.

**Figure 4:**
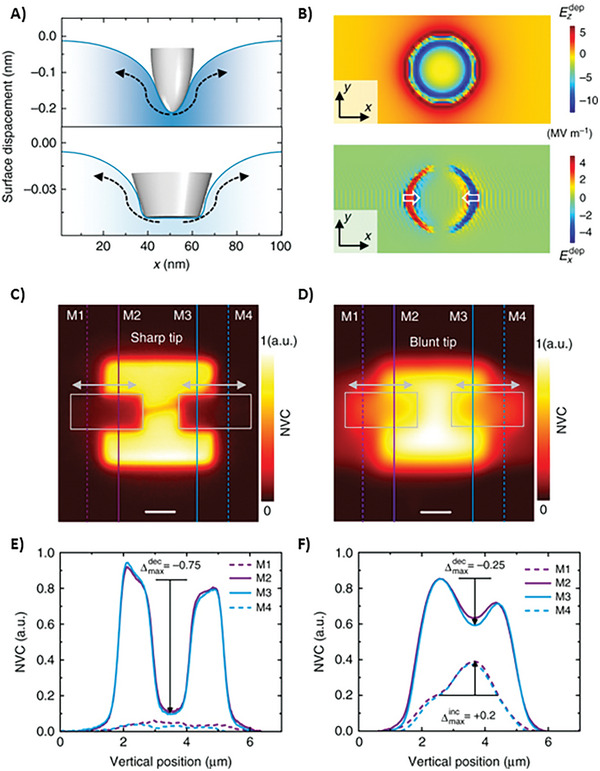
Controlled manipulation of oxygen vacancies. A) Simulated surface deformation profiles under a spherical (upper panel) and flat‐ended (lower panel) tip for a static contact force of 4 µN. B) Simulated in‐plane distribution of the *z*‐component, $E_{z}^{dep}$(upper panel) and *x*‐component, $E_{x}^{dep}$ (lower panel) of the depolarization field induced by the flat‐ended tip. The ripples in $E_{x}^{dep}$ are numerical artifacts. The normalized vacancy concentration (NVC) maps after mechanical scans were performed using a C) sharp and D) blunt tip with a contact force of 9.5 µN within the gray‐colored boxes. The horizontal arrows indicate the corresponding fast‐scan direction. Before the mechanical scans, Vo‐enrichment was performed by poling the pristine surface with a tip bias of −5 V. E,F) NVC profiles along lines M1, M2, M3, and M4 in c (E) and d (F). The scale bar in (C, D) represents 1 µm. Image adapted with permission from reference.^[^ ^124^ ^]^ Copyright 2017, Springer Nature.

Next, flexoelectricity was used to control oxygen vacancies by mechanical force from a scanning probe microscope (SPM) tip.^[^ ^124^ ^]^ The combined experimental and theoretical approach recently demonstrated that oxygen vacancies can migrate under a depolarization field generated by a stress gradient. This nanoscale flexoelectric effect allows for controlled spatial modulation of vacancies. Consequently, the scanning probe tip deterministically rearranged the spatial distribution of vacancies. While vacancies can migrate vertically under the influence of the tip, the depolarization field traps them around the contact edge. They found that the depolarization field could be tailored to permit preferred lateral transmission by changing the SPM tip geometry (as shown in **Figure** **4**).

### 2.5 Metals

Flexoelectric response in ferroelectrics is significantly larger than that of other materials. However, most of the known ferroelectric materials are in a nonmetallic state. Here, the response of metals to the flexoelectric effect is presented. Metals are known not to exhibit ferroelectricity, caused by conducting electrons that screen the internal electric field.^[^ ^125^ ^]^ Yurkov and Yudin proposed a simple model to verify the possibility of measuring the flexoelectric effect in metals. The authors made several assumptions: as a theoretical example, an aluminum rod was considered, and conduction electrons were treated as an ideal Fermi gas (charge is distributed all over the crystal). They assumed that the flexoelectric effect in metals is nothing else but a macroscopic electric field wave (polarization wave) propagating over it. The effect of localized electrons was neglected. The authors additionally proposed a method to observe the flexoelectric effect in metals using two electrodes, which would not be directly connected to the sample. The flexoelectric effect (polarization wave) is generated by the collision of the two metallic rods. It has also been suggested that flexoelectricity in metals is a bulk property of the material; hence, it is highly dependent on the geometry of the sample. Still, according to these authors, there are many uncertainties as to why the propagation of an elastic wave generates charges that are only caused by the so‐called “flexo effect” in metals. The authors summarized the results by comparing the magnitude of the flexoelectric effect in metals to that of high‐*k* dielectrics.^[^ ^126^ ^]^

However, the prediction of metallic materials that exhibit ferroelectricity was made almost 60 years ago,^[^ ^127^ ^]^ and since then, it has been confirmed experimentally.^[^ ^128^, ^129^ ^]^ These are called polar metals and are primarily based on the composition of LiOsO_3_.^[^ ^130^ ^]^ They undergo a specific ferroelectric‐like phase transition and exhibit exceptional physical properties such as high optical birefringence and nonlinear susceptibility. Polar metals are promising for many applications, especially in optics and optoelectronics.^[^ ^131^ ^]^ For instance, TaAs have a giant nonlinear optical coefficient, showing a better response when aligned to the polarization.^[^ ^132^ ^]^ Zabalo and Stengel theoretically studied lithium osmate for its flexocoupling coefficient and found that the effective value in [100], [110], and [111] directions was 40.1, 5.3, and 2.5 eV, respectively.^[^ ^133^ ^]^

### 2.6 Strontium Ruthenate

SrRuO_3_ belongs to the ABO_3_ oxide perovskite group. It crystallizes in orthorhombic symmetry with *Pbnm* space group and lattice parameters of *a* = 5.53 Å, *b* = 5.57 Å, and *c* = 7.85 Å; however, the orthorhombic unit cell consists of four units, which results in an ideal perovskite structure with a lattice constant of 3.93 Å. Over 547 °C, it undergoes the phase transition into the tetragonal *I4/mcm* phase, and over 677 °C into the cubic *Pm3m* phase.^[^ ^134^ ^]^ SrRuO_3_ is also the first reported oxide with ferromagnetic properties.^[^ ^135^ ^]^

A recent theoretical study of flexoelectricity followed by experimental measurement in metallic oxides was made by Peng et al. The authors focused on SrRuO_3_ and compared their theoretical results with those of BTO and STO. Scanning transmission electron microscopy with an annular bright field (which can directly visualize the atomic structure) was used to investigate the displacement of Sr in the structure. Displacements of Ru in the initial centrosymmetric structure and strong polarization caused by the unit‐cell electrical dipole were observed. Subsequently, the structural symmetry was further investigated by optical second harmonic generation, followed by higher‐temperature measurements. First, this could distinguish three monoclinic domains with polar axes along [110], [101], and [011], which confirmed the presence of the polar monoclinic phase in the bulk of the STO films and the rhombohedral structure at the interface. When annealed, the transition from the polar to nonpolar phase was observed in a temperature range of ≈630 K. (electrostrictive‐like lattice expansion) caused by Ru off‐centering along the polar axis. This was the case that impacted the electronic properties of SrRuO_3_. Thus, the main conclusion is that polar metals can induce and control flexoelectricity directly.

### 2.7 Hafnium Oxide

Hafnium oxide is a high‐k dielectric material with a large band‐gap energy of 5.6–5.8 eV, dielectric permittivity of *κ* = 16–25, thermal stability reaching 2780 °C and high energy barriers for electrons and holes.^[^ ^136^ ^]^ The most stable polymorph of HfO_2_ at room temperature and atmospheric pressure is so‐called M‐phase with a space group *P2_1_/c*. However, there is still several different known polymorphs, from which all have a center of symmetry, which deny the occurrence of the ferroelectricic effect in HfO_2_.^[^ ^137^ ^]^

Fluorite HfO_2_‐based materials, which are known to be used in random access memories as high‐k materials, were investigated.^[^ ^138^ ^]^ The results showed that polarization in Hf_0.5_Zr_0.5_O_2_ thin films can be mechanically switched by applying the strain gradient (using piezoresponse force microscopy tip), which was additionally confirmed with the appearance of domain walls. The remanent polarization was in a range of 16 *×* 10^−6^ C cm^−2^. Indeed, Zakusylo and coworkers have shown that large magnetoelectric coupling in Co/Hf_0.5_Zr_0.5_O_2_ structures, with variations of internal electric fields of ≈100 kV cm^−1^ under magnetic field.^[^ ^139^ ^]^ Authors discussed that the large variation of internal electric field results from flexoelectric effects and that the flexoelectric coefficient of Hf_0.5_Zr_0.5_O_2_ must be larger than that of archetypical ferroelectrics as BaTiO_3_, as supported by DFT and analytical calculations. These results demonstrate the possibility of future application of (Hf, Zr)O_2_‐based thin films in flexoelectric‐based devices. Recently, the flexoelectricity of HfO_2_ in amorphous form was also tested. The measured flexoelectric coefficient was in a range of 105 ± 10 *×* 10^−12^ C m^−1^.^[^ ^140^ ^]^

### 2.8 Titanium Dioxide

**Figure 5:**
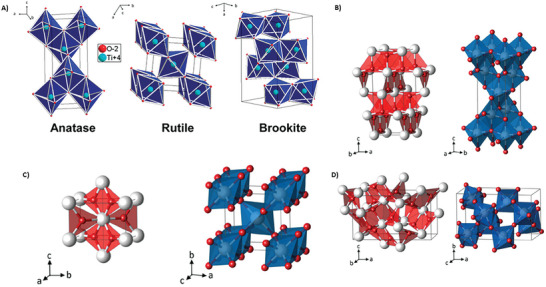
A) Representations of the TiO_2_ anatase, rutile, and brookite forms, Image taken with permission from reference.^[^ ^143^ ^]^ Copyright 2010, American Chemical Society. Planar Ti_3_O building‐block representation (red) and TiO_6_ polyhedra (blue) for B) the TiO_2_ phases anatase, C) rutile, and D) brookite (Ti (white); O (red)). Image taken with permission from reference.^[^ ^146^ ^]^ Copyright 2012, IOP Science

**Figure 6:**
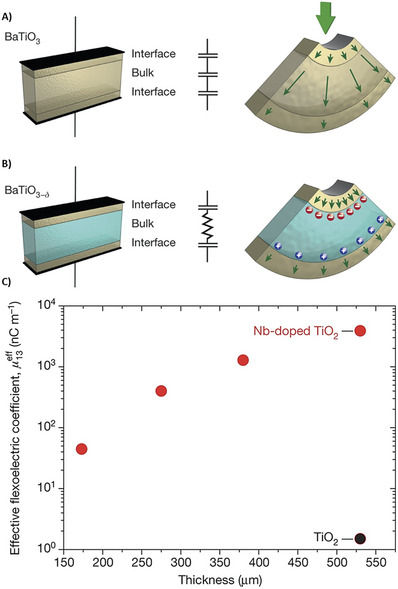
A) A dielectric with one bulk and two interfacial layers reacts to voltage like three capacitors in series. Similarly, polar contributions (sketched as green arrows) from bulk flexoelectricity and surface piezoelectricity are present in its electromechanical response to bending. B) At the interfaces where a semiconductor and electrodes meet, depletion layers may form. In these cases, the conducting bulk functions as an intercalated electrode (blue layer) between interfacial barrier layers, which react like thin capacitors. Larger capacitance (inversely proportional to barrier thickness) and increased surface piezoelectricity are the results of free charges (sketched as blue and red spheres) screening the internal depolarizing field. C) When bending Nb‐doped TiO_2_ (filled red circles) in two crystals of the same thickness, the change produced is three orders of magnitude greater than when bending the insulator (TiO_2_; black filled circle). A barrier‐layer model is consistent with the semiconductor's effective flexoelectric coefficient $\mu_{13}^{eff}$, which is proportional to sample thickness. Image adapted with permission from reference.^[^ ^108^ ^]^ Copyright 2016, Nature.

Currently, TiO_2_ is one of the most investigated photocatalytic materials^[^ ^141^ ^]^ and is known for its flexoelectricity, high permittivity, and nonferroelectricity or paraelectricity, which results in no hysteresis or saturation effects. It is also considered an environmentally friendly material because it contains no lead, unlike the industrially used ones.^[^ ^47^ ^]^ TiO_2_ naturally crystallizes in two tetragonal phases, anatase and rutile.^[^ ^142^ ^]^ The third, less common orthorhombic phase of TiO_2_ is brookite (metastable). The difference can be clearly seen in their octahedral structure, which shares only octahedral edges in anatase (**Figure** **5**A,B), while in rutile (Figure 5A,C) and brookite (Figure 5A,D), both corners and edges are shared.^[^ ^143^ ^]^ According to flexoelectricity, rutile is the preferred phase owing to its higher permittivity, which is the double permittivity of anatase.^[^ ^144^ ^]^ The static dielectric constant of rutile was measured as a function of temperature and was 257 and 111 at low temperatures, 170 and 86 at room temperature, and 97 and 58 at 1000 K in the *a* and *c* directions, respectively.^[^ ^145^ ^]^ Single‐crystal measurements were performed for TiO_2_ and Nb‐doped TiO_2_, clearly showing that Nb‐doped TiO_2_ has a much better flexoelectric response, which can be explained by the battier‐layer mechanism (**Figure** **6**A,B), and the effective flexoelectric coefficient was in the scale of 10^−6^ to 10^−5^ C m^−1^ (Figure 6C); hence, the authors did not mention the direction of the flexoelectric coefficient measured.^[^ ^108^ ^]^

Moreover, the flexoelectric coefficient increases with thickness (Figure 5C), which is contrary to the flexoelectric bulk mechanism rule but in accordance with the barrier‐layer mechanism mentioned above. In another study, polycrystalline samples were also subjected to testing, but when speaking of polycrystalline samples, it is impossible to estimate the flexoelectric coefficient for a specific direction.

Thus, the authors assumed that the measured flexoelectric coefficient combines longitudinal and transverse flexoelectric coefficients. The measured value was still in the 1.67 *×* 10^−9^ C m^−1^ in high‐frequency noises to 1.98 *×* 10^−9^ C m^−1^ in low‐frequency noise. It was also noticed that the measured flexoelectric coefficient was directly proportional to the length of = the measured cantilever.^[^ ^47^ ^]^ For single crystals of TiO_2_, the effective flexoelectric coefficient is in the range of 1–10 *×* 10^−9^ C m^−1^.^[^ ^147^ ^]^ Additionally, a clear photovoltaic response induced by force (the so‐called flexophotovoltaic response) for TiO_2_ single crystals was reported under laser light illumination with a wavelength of 405 nm.^[^ ^15^ ^]^

In semiconductors, depletion layers form at interfaces with electrodes. The conducting bulk acts as an intercalated electrode between interfacial barrier layers functioning as thin capacitors. This yields increased capacitance inversely proportional to barrier thickness and enhanced surface piezoelectricity due to the screening effect of free charges.

**Figure 7:**
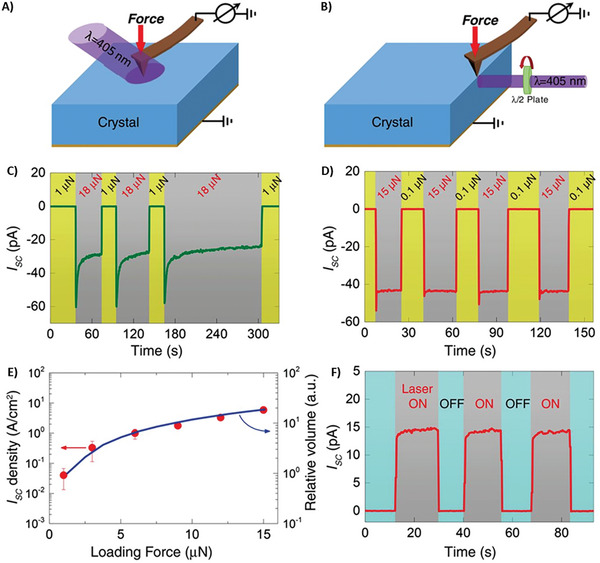
PV effects induced by force in centrosymmetric single crystals of SrTiO_3_ and TiO_2_. A) Setup the area associated with the contact area for illumination. The AFM's feedback loop was used to regulate the tip loading force. B) Configuration to light the side surface. To prevent Fresnel reflection and ensure that light absorption was unaffected by light polarization, this illumination geometry was selected. The photocurrent evolution on C) a SrTiO_3_ (001) face and D) a TiO_2_ (100) face, respectively, was induced and collected by a conductive AFM tip with a high loading force. E) Loading force dependence of the relative volume and induced photocurrent density under a strain gradient greater than 1 × 106 m^−1^. F) TiO_2_ (001) was used to measure positive photocurrent. Image adapted with permission from reference.^[^ ^15^ ^]^ Copyright 2018, Science.

Recent findings show that photovoltaic mechanisms that allow solar cells to operate at higher efficiency are exceptionally desirable.^[^ ^15^ ^]^ Silicon can realize the bulk photovoltaic effect by mediating the flexoelectric effect, as shown in **Figure** **7**A–F. This effect is free from the thermodynamic Shockley–Queisser limit and typically manifests only in noncentrosymmetric (piezoelectric or ferroelectric) materials. Centrosymmetric single crystals of strontium titanate, titanium dioxide, and silicon were subjected to strain gradients created by either an atomic force microscope or a micrometer‐scale indentation system. This resulted in the generation of substantial photovoltaic currents. This bulk photovoltaic effect, referred to as the flexophotovoltaic effect, is activated without a p–n junction by strain gradients. The finding can potentially improve current solar cell technologies by increasing solar energy conversion effectiveness. In recent years, authors reported an increase in photocurrent in proton‐doped, hydrogen‐charged TiO_2_ (rutile) crystals of over two orders of magnitude. Proton doping was responsible for inducing the presence of charge carriers and expanding/distorting the crystal lattice, generating a strain gradient. The formation of the O–H bonding also led to a centrosymmetry break, which, when combined with proton doping, resulted in a significant flexoelectric field. Additionally, when proton doping was used on a single crystalline rutile, photocurrent was increased by three orders of magnitude.^[^ ^148^ ^]^

When speaking of catalysis, nanoscaled titanium dioxide (TiO_2_) was immobilized by polyvinylidene fluoride (PVDF) beads through the phase inversion method, which led to a highly efficient photocatalytic degradation of ciprofloxacin under ultraviolet light. This efficiency was demonstrated by the elimination of the antibacterial activity of the solution following degradation. Moreover, the stability of the prepared beads remained intact even after 30 cycles, with only a minor reduction in efficiency, which was attributed to the accumulation of trace amounts of organic pollutants.^[^ ^149^ ^]^ In recent years, TiO_2_ was also used as a filler in a nanocomposite based on polypropylene, which caused an energy density increase of almost 175% when compared to pure polypropylene films. These types of composite can be further used as dielectric materials or in energy storage systems.^[^ ^150^ ^]^ Flexocatalysis in nanoscaled TiO_2_ (rutile) shows great potential in green and biocompatible catalysis. In recent work, authors have developed a complex flexocatalytic mechanism that marks that polarization under ultrasound simulation can reduce the exhaustion of reaction species on the catalyst's surface, which greatly enhances its efficiency. What is more, the flexoelectric polarization is switchable, and in contrast to the switching in ferroelectrics with piezoelectric properties, the flexoelectric polarization in nonferroelectric materials has no high energy barrier. This leads to the conclusion that flexoelectric‐based catalysis (flexocatalysis) is, in fact, much more efficient. It was noted, that the correlation between particle size and strain gradient in flexoelectricity can be established by the simple constant scale factor, which can only be influenced by the specific crystallographic orientation.^[^ ^151^ ^]^

### 2.9 Zinc Oxide

Zinc oxide has been known as a material with strong piezoelectric and weak ferroelectric responses.^[^ ^152^, ^153^ ^]^ ZnO is proposed to be the first ferroelectric with electronic origin, which nowadays is a widespread topic of investigation,^[^ ^154^ ^]^ with the ISI database reaching about 50 thousand papers. It is an II–VI wide‐gap compound semiconductor with an exciton binding energy of 60 meV and a bandgap of around 3.4 eV.^[^ ^155^ ^]^ It crystallizes in the specific hexagonal wurtzite lattice with a space group *P6_3_mc*, which is composed of two hexagonal close‐packed sublattices, known as a wurtzite‐type structure.^[^ ^156^ ^]^ It can be found as an extremely rare mineral – zincite.^[^ ^157^ ^]^ Additionally, it was shown that specific doping can modify its bandgap and improve its response.^[^ ^158^ ^]^ For instance, when doped with vanadium (V), it has a giant piezoelectric response, with a piezoelectric coefficient reaching 110 pC N^−1^, showing its possibility for application in piezoelectric devices.^[^ ^159^ ^]^ Other studies show that with Li‐ doping, an enhancement of remanent polarization was observed.^[^ ^160^ ^]^ In the form of nanowires, ZnO was used back in 2006 as piezoelectric nanogenerators. In bent state, the nanowire reached an efficiency of about 30%.^[^ ^41^ ^]^ Despite of the low voltage in a single ZnO nanowire (≈20–50 mV),^[^ ^161^ ^]^ nowadays, ZnO has been used in heterostructures as a flexoelectricity‐enhanced piezoelectric nanogenerator, which was based on Zn–Al‐layered hydroxide nanosheets – ZnO heterostructures. The achieved output power density under tapping reached 2.7 µW cm^−2^.^[^ ^42^ ^]^ It is important to mention, that the weak fereoelectric response observed first in forest nanowires, was associated to strain and variations on the lattice constant, which induced the polarization of the system,^[^ ^162^ ^]^ nevertheless the weak response observed in freestanding particles, might be indeed associated to a relaxation effect between ferroelastic(flexoelectric) coupling and improper ferroelectricity,^[^ ^163^ ^]^ but the general presence of flexoelectricity might play a vital role in this effect.

Another study showed the effect of functionalization of ZnO‐nanowires by Au nanoparticles, and as proved, the photodetection performance was highly improved, and the pyroelectric field was also enhanced.^[^ ^164^ ^]^ In recent years, ZnO nanorods were additionally produced and used with success for wearable electronics, where the coupling of piezo‐ and flexoelectric effect of ZnAl‐layered nanosheets and ZnO‐nanorods was performed, resulting in a maximum power density reaching 68.2 µW cm^−2^.^[^ ^165^ ^]^ The spherical ZnO nanoparticles are also known to be prominent photocatalytic material candidates because of, at first, their much broader range of light absorption when compared to TiO_2_ and, second, their much lower production cost_._^[^ ^166^ ^]^ Until today, there has been no accurately measured flexoelectric coefficient for ZnO to the best of our knowledge of literature.^[^ ^167^ ^]^

**Figure 8:**
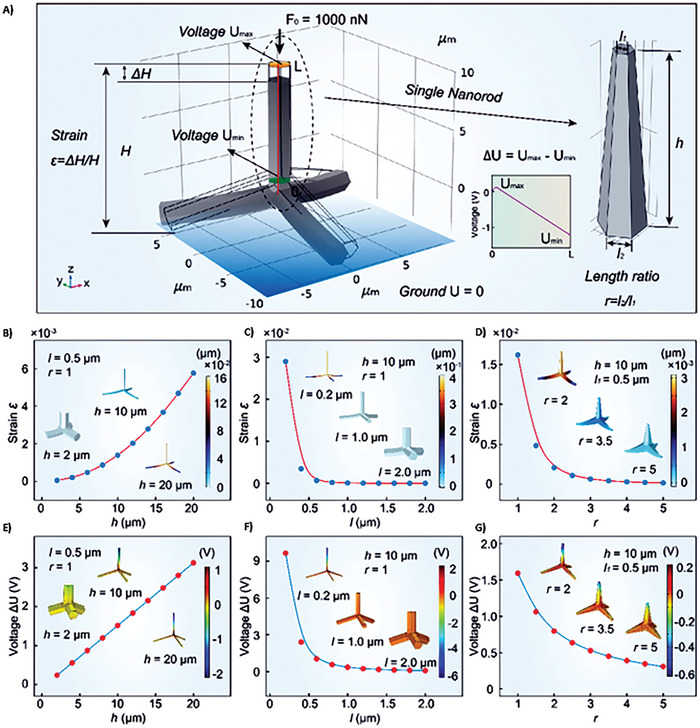
Finite element modeling simulations of the piezoelectric response of ZnO tetrapods. Height, length, and ratio of sides strongly influence the piezoelectric response. a) Simulation model of the ZnO tetrapod length, height, and side ratio (bottom to top length). b, e) strain and piezoelectric potential of the ZnO tetrapod with height h) varying from 2 to 20 µm). c, f) Strain and corresponding piezoelectric potential of the ZnO tetrapod with length (*l*) varying from 0.2 to 2.0 µm). d,g) The strain and piezoelectric potential of the ZnO tetrapod with length ratio (*r*) from 1.0 to 5.0). Reproduced with permission from AIP.^[^ ^181^ ^]^

However, the most promising and complicated form of ZnO currently used is the nanotetrapod (Section **5.2**). These structures are composed of four 1D ZnO nanorods interconnected together via a central core at an angle of around 105° with respect to each other, leading to a three‐dimensional (3D) geometry. The tetrapod structure retains the inherent physical and chemical features of ZnO and also benefits from the 3D tetrapod geometry. This configuration significantly enlarges their active surface area accessibility, enhancing their effectiveness when utilized collectively, as they are able to self‐assemble themselves into an interconnected chain‐like complex 3D network architecture with high porosity.^[^ ^168^ ^]^ The synthesis of ZnO tetrapods is already established using several methods, for instance: flame transport approach,^[^ ^169^ ^]^ vapor phase synthesis,^[^ ^170^ ^]^ hydrothermal process,^[^ ^171^ ^]^ synthesis using wet chemistry,^[^ ^172^ ^]^ thermal evaporation,^[^ ^173^ ^]^ evaporation and recondensation of metallic Zn,^[^ ^174^ ^]^ chemical vapor deposition,^[^ ^175^ ^]^ vapor phase growth,^[^ ^176^ ^]^ combustion method,^[^ ^177^ ^]^ and with the assistance of microwave plasma.^[^ ^178^ ^]^ However, flame transport synthesis is the most reliable method to produce the ZnO micro‐ and nanotetrapods in a reproducible manner and in large quantities. To follow the statement, their wide possible applications are, to name a few, as follows: electronics, photonics, lasing, clean energy production and nanogenerators, medical and biomedical (drug and gene transport/delivery, antimicrobial, antiviral, antifungal, etc.), ceramic and glass (according to this work – flexible ceramic materials), food production, and plant growth and fertilizers.^[^ ^169^, ^179^, ^180^ ^]^ A recent work reports, that the arm dimensions (length and diameters) in ZnO can be easily controlled via the temperature, concentration, oxygen inlet, and synthesis method – which significantly affect their mechanical and electrical properties.^[^ ^180^ ^]^ The one‐dimensional (1D) ZnO nanorods naturally exhibit the piezoelectric behavior and that has been investigated in detail by several groups. The tetrapod shape which is a 3D construction out of four 1D ZnO rods, is very interesting geometry from piezoelectric response point of view. The piezoelectric response mainly occurs due to the bending of arm under the external force which is easy to estimate in 1D, but due to the complex structure, the arms of tetrapods bent differently under the stress. The resultant piezoelectric response in tetrapods is decided by the way arms bent and the resultant piezo‐potential developed. Understanding the piezoelectric response in ZnO tetrapods is definitely an extremely demanding undertaking. In a recent theoretical study, it has been reported that the aspect ratio of ZnO arms in the tetrapod play important role in their piezo response. The investigations clearly showed that the longer and thinner the ZnO nanorods are, the larger strain and voltage will be generated. When the arm is pressed from the top, the central core bents downwards leading to uniform bending of other three arms which most likely generates an enhanced output voltage^[^ ^181^ ^]^ (**Figure** **8**).

Furthermore, as ZnO is mostly known for its piezoelectric response, this response in tetrapods is not fully exploited unless anisotropic/isostatic pressure is applied to them. A further theoretical investigation has reported that the crystalline structure at the central core in ZnO tetrapods plays a crucial role in the piezo transport property. Under the influence of force, more charge carriers are generated, leading to improvement in tetrapods' semiconducting and transport properties.^[^ ^182^ ^]^ The combined piezoelectric and carrier enhancement phenomenon under the force (without breaking), makes these tetrapods very interesting for many applications and could lead to an interesting phenomenon of flexoelectricity, an essential aspect to be explored further. This might open several avenues toward using these tetrapods in interesting energy generation and simultaneously catalytic activities which are very much needed to drive photocatalytic water splitting. Due to the unique crystal structure of the central core in the three‐dimensional tetrapod, the arm bending leads to enhance the carrier concentration which can play a pivotal role in energy harvesting and catalysis, and the tetrapods need to be explored in detail for flexocatalysis.

### 2.10 Halides

This review will discuss only halide perovskites with archetype XPbI_3_ (where *x*‐methylammonium cation – CH_3_NH_3_^+^). These types of halides are already known in the industry for their solar cell applications, with a high efficiency exceeding 20%. However, their main disadvantage is their susceptibility to photodegradation.^[^ ^184^ ^]^ Additionally, halide perovskites exhibit a huge static dielectric constant,^[^ ^185^ ^]^ which can be attributed to their structural fluctuations in the perovskite cell, which are also enhanced by the possibility of rotation of the CH_3_NH_3_ cations.^[^ ^186^ ^]^ Methods of single‐crystal growth from solution have already been established.^[^ ^187^ ^]^ Recent studies of the effective flexoelectric coefficient in single crystals of XPbBr_3_ and XPbCl_3_ indicated that the values for both halides were similar, in the order of ≈3 *×* 10^−5^ C m^−1^. This is an immense value compared to other known flexoelectric materials, reaching even enormously high values of 1.7–2.2 *×* 10^−3^ C when the material is exposed to light.^[^ ^88^ ^]^ Authors mentioned that the bulk flexoelectric effect is most likely not able to reach such values; therefore, one should also remember the other effects existing in ionic conductors – like “the flexoionic effect”^[^ ^188^ ^]^ or the photoinduced compositional segregation or transport on the grain boundaries.

**Figure 9:**
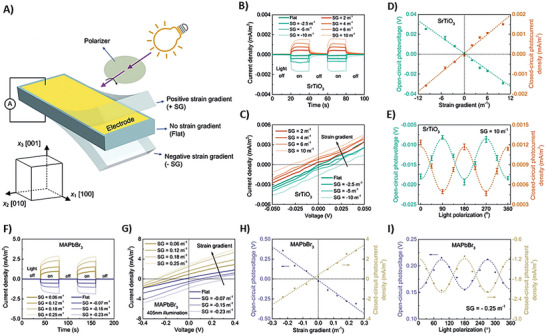
Schematic setup for the flexophotovoltaic experiment and outcomes for SrTiO_3_ (STO) and MAPb crystals are detailed. A) Crystals with matching top and bottom electrodes are secured in the thickness direction [001] and vertically bent around the [010] axis, with lateral illumination. A polarizing filter postmeasurements ensures confirmation of the bulk nature of the photoresponse, conducted under 1000 LUX illuminance. B, F) Illustrate photocurrent density concerning applied strain gradient. C, G) Depict current as a function of voltage during bending and illumination. D, H) Open‐circuit photovoltage and closed‐circuit photocurrent as functions of strain gradient, extracted from (C) and (G). E, I) Explore photovoltage and photocurrent concerning incident light polarization relative to the [001] direction, perpendicular to electrodes and parallel to flexoelectric polarization. The observed π‐periodic response aligns with a bulk photovoltaic effect. Image taken with permission from the reference.^[^ ^183^ ^]^ Copyright 2024 American Physical Society.

Wang et al. conducted a flexophotovoltaic experiment investigating STO and MAPb crystals. In **Figure** **9**A, crystals with identical electrodes were vertically bent around the [010] axis and illuminated laterally under 1000 LUX. A polarizing filter confirmed the bulk nature of the photoresponse. Photocurrent density, shown in Figure 9B,F, revealed the correlation between strain and generated current. Figure 9C,G are focused on current under bending and illumination, detailing electrical characteristics. Open‐circuit photovoltage and closed‐circuit photocurrent in Figure 9D,H, derived from Figure 9C,G, respectively provided insights into material response to varying strain. Exploring incident light polarization in Figure 9E,I, perpendicular to electrodes and parallel to flexoelectric polarization, a consistent π‐periodic response indicated a bulk photovoltaic effect. The legends in each graph are crucial in facilitating interpretation, ensuring a systematic and clear presentation of the experiment's configuration and results.^[^ ^183^ ^]^ This study enhances our understanding of flexo photovoltaic phenomena and their potential applications in photovoltaic technologies. In addition, theoretical studies of elastic dielectrics with polarization gradients using a lattice‐dynamic approach were performed for several halides, such as NaI, NaCl, KI, and KCl. The total potential of the ionic polarization was determined using the shell model. Elastic constants were estimated to be 0.359 *×* 10^12^, 0.486 *×* 10^12^, 0.27 *×* 10^12^, and 0.4 *×* 10^12^ dyn cm^−2^ in NaI, NaCl, KI, and KCl, respectively.^[^ ^189^ ^]^ Other studies of NaCl and KCl with the shell model gave flexoelectric constant values of 0.412 *×* 10^−13^, −0.122 *×* 10^−13^, and −0.212 *×* 10^−13^ C m^−1^ for µ_11_, µ_12_, and µ_44_ NaCl, and 0.403 *×* 10^−13^, −0.122 *×* 10^−13^, and −0.228 *×* 10^−13^ C m^−1^ for µ_11_, µ_12_, and µ_44_ KCl.^[^ ^56^ ^]^

### 2.11 Dichalcogenides and Others

Sulfides and dichalcogenides are commonly used materials for energy harvesting.^[^ ^190^, ^191^, ^192^, ^193^ ^]^ For instance, FeS_2_ devices have already been investigated as hybrid electrodes, where carbon‐covered nanoparticles have a power conversion efficiency of over 8%.^[^ ^194^ ^]^ Additionally, in dichalcogenides, such as MoS_2_, MoSe_2_, MoTe_2_, and WS_2_, when applying an electric field, their band gap could be decreased, even reaching the metallic state.^[^ ^195^ ^]^ These materials would be promising for photoflexoelectricity, because they have an easily tunable bandgap, especially in 2D materials.^[^ ^196^ ^]^ Speaking of the flexoelectricity, the MoS_2_ effective flexoelectric coefficient measured was ≈0.1 *×* 10^−9^ C m^−1^.^[^ ^197^ ^]^ In the case of ZnS, it has also been theoretically investigated. The shell model lattice dynamics calculations yielded different values of –0.311 *×* 10^−13^, –1.544 *×* 10^−13^, and –0.611 *×* 10^−13^ C m^−1^ for µ_11_, µ_12_, and µ_44_, respectively.^[^ ^56^ ^]^ The detailed values are presented in **Table** **1**.

**Table 1:** Measured and calculated flexoelectric coefficient values for various flexoelectric materials. The direction of the flexoelectric coefficient and methods have been listed, when available from their source.

Material	Coefficient	Flexoelectric constant [C m^−1^]	Method	Refs.
SrTiO_3_	[100][010][001]	6.1 × 10^−9^		
SrTiO_3_	[10–1][010][101]	−5.1 × 10^−9^	Dynamic mechanical analyzer	[58]
SrTiO_3_	[11–2][−110][111]	−2.4 × 10^−9^		
SrTiO_3_	[100][010][001]	12.4 ± 0.6 × 10^−9^	The three‐point bending approach	[198]
SrTiO_3_	[110][−110][001]	−8.3 ± 1.4 × 10^−9^		
SrTiO_3_		1.1 ± 0.1 × 10^−9^	Cantilevered beam‐based approach	[199]
BaTiO_3_		18.5 × 10^−9^	Molecular dynamic simulations	[200]
BaTiO_3_	Orthorhombic‐ tetragonal	5‐50 × 10^−9^	Cantilever beam‐based approach	[107]
BaTiO_3_	µ_12_ (T_c_ + 3.4 K)	≈50 × 10^−6^	Cantilever bending	[201]
Ba_0.67_Sr_0.33_TiO_3_		up to 100 × 10^−6^	Cantilever bending	[202]
Ba_0.67_Sr_0.33_TiO_3_		150 × 10^−6^	Pyramid compression	[203]
Ba_0.67_Sr_0.33_TiO_3_	µ_11_	150 × 10^−6^	Pyramid compression (0.5 Hz)	[200, 201]
Ba_0.67_Sr_0.33_TiO_3_	µ_11_	120 × 10^−6^	Converse flexoelectric effect (400 Hz)	[201]
Ba_0.67_Sr_0.33_TiO_3_	µ_12_	100 × 10^−6^	Cantilever bending (1 Hz)	
Ba_0.65_Sr_0.35_TiO_3_	µ_12_	8.5 × 10^−6^	Cantilever bending	
BST/Ni_0.8_Zn_0.2_Fe_2_O_4_		128 × 10^−6^	Cantilever beam‐based approach	[204]
PVDF		0.016 × 10^−6^	Cone‐shape material compression	[205]
PVDF		3.17 ± 0.41 × 10^−9^		
PVDF with 10% BST		4.36 ± 0.93 × 10^−9^		
PVDF with 15% BST		6.81 ± 0.70 × 10^−9^	Dynamic mechanical analyzer	[117]
PVDF with 20% BST		8.77 ± 1.19 × 10^−9^		
PVDF with 25% BST		13.50 ± 1.76 × 10^−9^		
TiO_2_	[100][010][001]	1.7 ± 0.3 × 10^−9^	The three‐point bending approach	[198]
TiO_2_	Longitudinal and transverse	1.65 – 1.98 × 10^−9^	The three‐point bending approach	[108]
0.05Nb doped TiO_2_		10^−6^ to 10^−5^		
NaCl	µ_11_	0.412 × 10^−13^		
NaCl	µ_12_	−0.122 × 10^−13^		
NaCl	µ_44_	−0.212 × 10^−13^	Estimated with empirical lattice dynamics	[56]
KCl	µ_11_	0.403 × 10^−13^		
KCl	µ_12_	−0.122 × 10^−13^		
KCl	µ_44_	−0.228 × 10^−13^		
MoS_2_		0.1 × 10^−9^	Piezoresponse force microscopy	[197]
ZnS	µ_11_	−0.311 × 10^−13^		
ZnS	µ_12_	−1.544 × 10^−13^		
ZnS	µ_44_	−0.611 × 10^−13^		
GaAs	µ_11_	0.8512 × 10^−13^	Shell model lattice dynamic calculation	[56]
GaAs	µ_12_	0.5107 × 10^−13^		
GaAs	µ_44_	0.1702 × 10^−13^		
GaP	µ_11_	0.4653 × 10^−13^		
GaP	µ_12_	0.3128 × 10^−13^		
GaP	µ_44_	0.3443 × 10^−13^		
XPbBr_3_		24 × 10^−6^	Piezoelectric actuator	[88]
XPbCl_3_		33 × 10^−6^		
PbMg_0.33_Nb_0.67_O_3_	µ_12_	3–4 × 10^−6^	Cantilever bending	
PMN‐PT	µ_11_	6–12 × 10^−6^	Pyramid compression (4‐10 Hz)	
PMN‐PT	µ_11_	20–50 × 10^−6^	Pyramid compression (0 Hz)	[201]
Pb_0.3_Sr_0.7_TiO_3_	µ_11_	20 × 10^−6^	Pyramid compression (0.5 Hz)	
Pb(Zr,Ti)O_3_	µ_12_	0.5 × 10^−6^	The four‐point bending approach	
Pb(Zr,Ti)O_3_	µ_12_	1.4 × 10^−6^	Cantilever bending (1 Hz)	
(Pb, Zr) TiO_3_		1.4 × 10^−9^		[206]
Pb(Mg_0.33_Nb_0.67_) O_3_		4.0 × 10^−6^	Cantilevered beam‐based approach	[207]
KTaO_3_	[100][010][001]	4.4 ± 0.5 × 10^−9^		
YAlO_3_	[101][10–1][010]	−3.7 ± 0.2 × 10^−9^	The three‐point bending approach	[198]
DyScO_3_	[101][10–1][010]	−8.4 ± 0.4 × 10^−9^		
LaAlO_3_	[100]_pc_ [010]_pc_ [001]_pc_	3.2 ± 0.3 × 10^−9^		
Hydroxyapatite		6 ± 1 × 10^−9^	Cantilevered beam‐based approach	[199]
Amorphous HfO_3_		105 ± 10 × 10^−12^	Laser Doppler Vibrometer‐based approach	[140]

**Figure 10:**
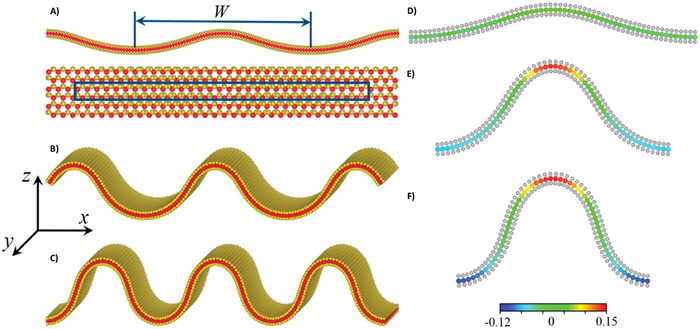
A–C) Relaxed structures of the wrinkled MoSe_2_ monolayers at wavelengths of 6.6, 8.0, and 9.8 in top and side views, respectively. The blue rectangle shows the unit cell in (A), and *W* is the wavelength following wrinkling, which is equal to the unit cell's length. The red and yellow dots represent the Mo and Se atoms, respectively, and periodic boundary conditions are applied in both the *x* and *y* directions. Plotting contours of the Mo atoms' atomic dipole moments (measured in e Å) for wrinkled MoSe_2_ monolayers at D) 9.8 nm, E) 8.0 nm, and F) 6.6 nm in wavelength. Image taken with permission from the reference.^[^ ^209^ ^]^ Copyright 2024 American Physical Society.

Theoretical density functional theory (DFT) calculations made for several sulfates and dichalcogenides also confirmed that their thin monolayers (especially in MoS_2_), when wrinkled, generate a dipole moment inside the layer (**Figure** **10**A–F), which is higher when structural symmetry is decreased. However, the total polarization is a combination of effects, such as flexoelectricity, structural symmetry, and induced piezoelectricity. Another theoretical study, based on the Landau–Ginzburg–Devonshire free energy equation (commonly used for paraelectrics), showed that flexoelectricity can be responsible for the induction of polarity displacement in dichalcogenides. Additionally, an external electric field reversed this out‐of‐plane polarization induced by flexoelectricity in spontaneously rippled thin layers.^[^ ^208^ ^]^

### 2.12 Layered Double Hydroxide

**Figure 11:**
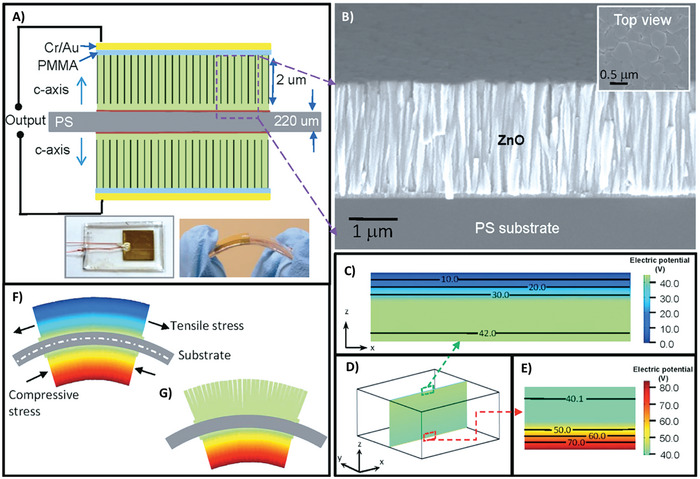
Fabrication of nanogenerators and their operation. A) The nanogenerator's manufacturing process. Images of the constructed nanogenerator after packaging are shown in the lower‐right sections. This resulted in good mechanical flexibility from the nanogenerator being bent. A cross‐sectional SEM image B) shows as‐grown nanowire textured film on substrate. A view of the nanowire film from above is displayed in the inset. D) The simulation model is used to study the operation of nanogenerators. A flexible substrate and ZnO films are present on a triple‐layer rectangular box's top and bottom surfaces. As indicated by C) and E), respectively, are the computed local potentials across the top and bottom electrodes. F) Spread of the piezopotential in the planned structure if the ZnO NWs create a solid, tightly packed film and exhibits continuous medium. The white dashed line in the substrate shows the strain‐neutral plane. G) Distribution of piezopotential in the designed structure: NWs on the stretched side of the substrate may not contribute to the output voltage, whereas NWs on the compressed side always result in a piezopotential drop. This is because ZnO NWs form a densely packed film, but there is a chance of small gaps or interwire sliding. Image taken with permission from the reference.^[^ ^212^ ^]^ Copyright 2024 American Physical Society.

Zinc/aluminum‐layered double hydroxides nanosheets were investigated for their flexoelectric properties, where the flexoelectric coefficient was estimated to be 1.8 (± 0.35) *×* 10^−6^ C m^−1^.^[^ ^210^ ^]^ The nonferroelectric composite (Bi_1.5_Zn_0.5_)(Zn_0.5_Nb_1.5_)O_7_/Ag flexoelectric coefficient was 1.7 *×* 10^−7^ C m^−1^ at room temperature.^[^ ^211^ ^]^ Moreover, ZnO nanowire films have been proven effective as low‐frequency mechanical energy harvesters with an output voltage reaching 10 V and a current over 0.6 *×* 10^−6^ A.^[^ ^212^ ^]^ The authors did not mention flexoelectricity (just flexible electronics), but according to the schematically presented experiment (**Figure** **11**), The fabrication of nanogenerators and their operational dynamics are crucial aspects in advancing energy harvesting technologies. Figure 11A illustrates the meticulous manufacturing process, where images reveal the final nanogenerator's mechanical flexibility, showcased through bending. Cross‐sectional scanning electron microscopy (SEM) imagery (Figure 11B) provides insights into its structural integrity. Utilizing simulation models (Figure 11D) aids in understanding nanogenerator operation, with flexible substrates housing ZnO films facilitating the study of local potentials across electrode surfaces. The planned structure's piezopotential spread is elucidated through Figure 11F–G, emphasizing the significance of solid, tightly packed ZnO nanowire films for continuous energy generation and potential disparities in output voltage due to variations in substrate strain. These findings underscore the intricate interplay between fabrication techniques and operational characteristics, guiding the advancements in nanogenerator design as confinement and nanostructurization can lead to a higher energy density and, thus, efficiency energy harvesting applications, which might be more important parameters than direct/discreet electrical outputs.

## 3 Methods of Direct Study

The flexoelectric effect can be directly measured using experimental techniques and methods that measure a material's flexoelectric response. The response may strongly depend on the measurement orientation and is related to the anisotropic properties of the tested sample, like the crystallographic orientation of the material or the shape of the sample. Hence, the flexoelectric response can be measured transversely, longitudinally, or sheerly.^[^ ^213^ ^]^

The primary method to measure the transverse flexoelectric coefficient is based on cantilever bending experiments. In this approach, the electric polarization is induced in the bending region of the cantilever, where the strain gradient appears. Similarly, the longitudinal coefficient is obtained from a change in electrical polarization induced by applying a compressive or tensile force to the sample. The opposite method is determining the shear coefficient, in which the applied voltage causes the sample to become electrically polarized and induces a strain gradient.

Currently, the methods used for the direct study of the flexoelectric effect generally involve techniques such as bending tests (e.g., cantilevers),^[^ ^87^, ^199^ ^]^ piezoresponse force microscopy, electric field microscopy, X‐ray diffraction, optical methods (interferometry, ellipsometry), dielectric microscopy, scanning Kelvin microscopy, or ferroelastic domain imaging. Because the effect is significantly more prominent at the micro/nanoscale, the experimental setups are generally based on scanning probe microscopy (SPM) techniques and utilize a small probe. Direct studies are commonly used for perovskites, single crystals, or polymers.^[^ ^87^ ^]^

### 3.1 Dynamical Mechanical Analyzer (DMA)

One method to study the flexoelectric effect directly is a dynamic mechanical analyzer (DMA).^[^ ^30^ ^]^ The experimental DMA setup (Figure 2A) measures the polarization induced by oscillatory bending, which causes a strain gradient in the sample. An insulating quartz probe applies the oscillating bending force while the sample is supported on two fulcrums. The DMA provides information based on the measured probe amplitude and current flow due to the induced polarization. Besides that, various mechanical properties, including loss modulus, storage modulus, and damping coefficient, can be determined from the DMA measurements. Additionally, the DMA setups usually allow for temperature‐dependent characterization. To extract the longitudinal or transverse flexoelectric coefficient (µ), the following Equation (3) is used:

(3) $$P_{3}=\mu \frac{\partial \varepsilon_{11}}{\partial x_{3}}$$

where *P* is an average polarization and the term ∂ɛ_11_/∂*x*_3_ stands for a strain gradient, which is mathematically derived from the following Equation (4):

(4) $$\frac{\partial \varepsilon_{11}}{\partial x_{3}}=\frac{12z_{0}}{L^{3}}\;\left(L-a\right)$$

Here, *L* is the distance between the fulcrums, *z*_0_ is the displacement measured by DMA, and *a* is the half‐length of the electrodes.

The flexoelectric coefficient of STO single crystal was measured using the described method.^[^ ^30^ ^]^ The experiment was performed with different crystallographic orientations, and an estimation of all flexoelectric tensors was provided. The measured values ranged from 1 *×* 10^−9^ to 10 *×* 10^−9^ C m^−1^. Moreover, by providing this method, the authors did not observe any piezoelectric or second harmonic generation response. Instead, the sign of local polarization around defects and dislocation with a value ranging from 1 *×* 10^−6^ to 10 *×* 10^−6^ C cm^−2^ was observed. This was explained by the possible polarization of the STO domain walls, such as the polarization through flexoelectricity, induced by the order‐parameter coupling between ferroelastic distortion and ferroelastic polarization. This effect could also be caused by domain walls trapping charged defects.^[^ ^30^ ^]^

Despite being a promising method, DMA has its limitations. The mechanical resilience of the samples directly limits the achievable oscillation amplitude. For instance, single‐crystalline samples might break when the amplitude exceeds a few micrometers. Additionally, generating submicrometric oscillations to induce small strain gradients is still impossible in such experimental setups, consequently resulting in a rather small window for measurements. Another limiting factor of the DMA is its maximum operational frequency, which is typically in the range of 10 Hz. Since higher frequencies generally provide improved signal detection, significantly lower frequencies might cause some problems, like not reaching the limit of detection of the signal.^[^ ^199^, ^214^ ^]^ Finally, a relatively large sample (on a millimetric scale) is required, similar to all the methods. However, these requirements are also met in every characterization technique induced by high‐frequency oscillatory bending.

### 3.2 Free‐Standing Cantilever Beam

**Figure 12:**
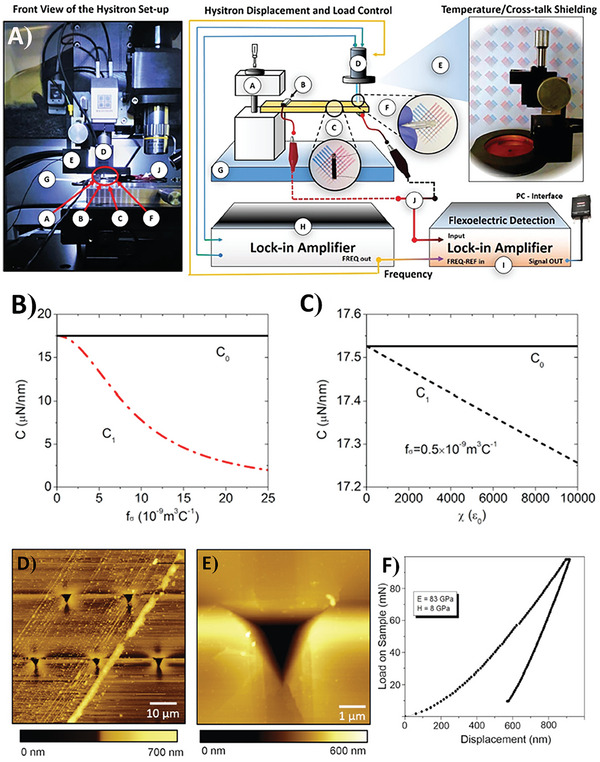
A) Frontal view and schematic illustration of the standard measurement setup. a) Stage mounting screw and Teflon clamps. b) Connecting the sample electrodes with platinum wire. c) A suspended cantilever beam displaying a sample of strontium titanate. d) The transducer head. e) The high temperature module's heat jacket. f) Berkovich tip, along with a picture of the actual probe. g) Stage of instruments. h) Lock‐in amplifier, which comes preconfigured with the tri‐indenter. j) Two‐way coaxial splitter is used to reconstruct the signal, and i) is a second lock‐in amplifier to detect the sample signals. Image taken with permission from the reference.^[^ ^199^ ^]^ Copyright 2020, Elsevier; phenomena associated with stiffness softening: B) Dependency of the flexoelectric coupling coefficient. C) Dielectric susceptibility dependence. Image taken with permission from reference.^[^ ^218^ ^]^ Copyright 2016, AIP Publishing. D) An AFM topography image measuring 70 µm × 70 µm showing the nanoindentation pattern. E) A typical topography map measuring 6 µm × 6 µm showing a nanoindentation. F) The associated load–displacement curve. Image taken with permission from the reference.^[^ ^73^ ^]^ Copyright 2019, Wiley.

**Figure 13:**
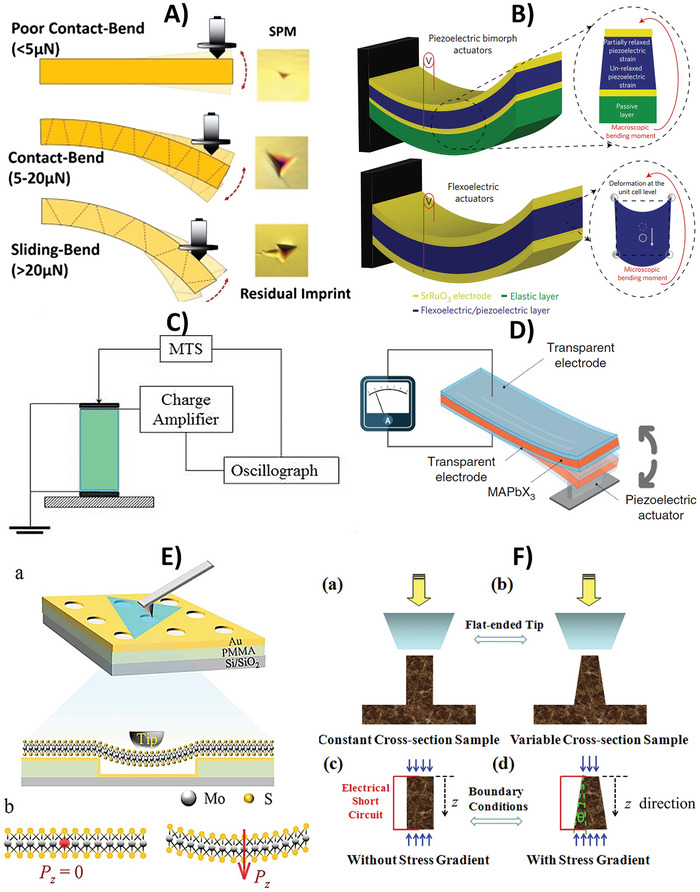
Experimental setups of direct and indirect measurement of the flexoelectric effect. A) Setup for direct flexoelectric measurements based on the nanoindentation method using free‐standing cantilever beams. Image taken with permission from reference.^[^ ^199^ ^]^ Copyright 2020, Elsevier B) Schematic comparison of flexoelectric and piezoelectric composite actuators based on a cantilever. Image taken with permission from reference.^[^ ^87^ ^]^ Copyright 2016, Nature C) Setup for indirect measurement of the flexoelectric effect based on nanocompressive testing. Image taken with permission from reference.^[^ ^205^ ^]^ Copyright 2016, AIP Publishing D) A piezoelectric actuator that delivers an oscillatory force to a cantilever‐shaped material. Image taken with permission from reference.^[^ ^88^ ^]^ Copyright 2020, Nature E) Setup for indirect flexoelectric measurement based on Piezoresponsive Force Microscopy (PFM). Image taken with permission from reference.^[^ ^216^ ^]^ Copyright 2019. Wiley F) Setup for indirect measurement of the flexoelectric effect based on the nanocompression test. Image taken with permission from reference.^[^ ^218^ ^]^ Copyright 2016, AIP Publishing.

Another method for measuring the flexoelectric response is to study a free‐standing cantilever beam (FCB).^[^ ^199^ ^]^ The experimental setup is based on a nanoindenter, a tool for measuring the mechanical properties of materials at the microscale. The main part of the equipment is a triboindenter equipped with an indentation probe with a diamond tip, a continuous dynamic mechanical property measurement device, and a temperature control stage (**Figure** **12**A). The experimental studies are performed on millimetric‐size rectangular cantilever beam samples. The flexoelectric polarization appears between two sides of the cantilever beam as a function of strain. The relation between polarization and strain is given by Equations (3) and (4). Usually, a side effect in the form of a residual imprint occurs during the mechanical tests. **Figure** **13**A shows a schematic illustration of a cantilever beam bending at different loads and the corresponding residual imprint.

This method was used to determine the flexoelectric response of commercially available STO and Hydroxyapatite (HAp).^[^ ^199^ ^]^ The contact load during the experiment ranged from 1 to 2 µN, and the obtained flexoelectric coefficient for the STO was 1.1 *×* 10^−9^ C m^−1^. As for the HAp, the coefficient was greatly influenced by additional surface phenomena such as parasitic piezoelectricity and polar regions. It has also been observed that surface roughness plays a significant role in small oscillations. This is particularly important when the oscillation and roughness are of the same order of magnitude. Therefore, the experiment required higher contact loads to reduce the unwanted surface effects.

In summary, using nanoindenters to study FCB is a powerful method with several advantages over conventional methods. It allows testing materials in dimensions unavailable for standard experimental setups. In particular, it provides high sensitivity demanded for millimetric‐size sample studies, where submillimetric oscillations and relatively small strain fields are limits.

### 3.3 Inverse Flexoelectricity

An alternative method for flexoelectric coefficient studies is the inverse method, which measures the bending curvature induced by the external electric field.^[^ ^87^ ^]^ The following Equations (5) and (6) provide the theoretical relationship between the curvature and the applied electric field:

(5) $$k=\frac{\mu V}{D}$$

(6) $$D=\frac{E*t^{3}}{12\left(1-v^{2}\right)}$$

where *k* is the curvature, *D* is the flexural rigidity (based on the Poisson ratio (*v*), Young's modulus (*E*), and thickness of the material (*t*)), *V* is the applied voltage, and µ is the flexoelectric coefficient. From both equations, it can be observed that the generated bending curvature is inversely proportional to the cube of the cantilever thickness (*t*). This means that the bending induced by the applied voltage is multiplied by the factor of 8 every time the thickness is halved, making this method suitable for directly measuring the flexoelectric properties of nanodevices. Additionally, inverse scaling with Young's modulus makes it well‐suited to characterize softer materials.^[^ ^87^ ^]^

An example of utilizing the inverse flexoelectricity method is studying the flexoelectric properties of micromechanical systems on silicon.^[^ ^87^ ^]^ The nanocantilevers of STO, an active layer sandwiched between two strontium ruthenate (SrRuO_3_) layers acting as electrodes, were fabricated using silicon‐compatible technology (Figure 13B). Applying an alternating voltage induces the cantilever into oscillations due to a bending moment from the strain gradient. The cantilever oscillations were observed using a digital holographic microscope in the stroboscopic mode. The experiment demonstrated the potential of flexoelectricity for creating lead‐free electromechanical actuators suitable for integration into silicon for MEMS and NEMS applications. This method also proved that the electromechanical capabilities of SrTiO_3_ devices are on par with or exceed those fabricated with ZnO.

### 3.4 Atomic Force Microscopy (AFM)

A different approach relies on atomic force microscopy systems, where an AFM probe exerts point force on a tested sample. The local inhomogeneous strain produced by the AFM probe at a point of contact breaks the centrosymmetric structure of the sample. Figure 9A,B schematically shows this. A conductive AFM probe is used to measure the sample electric response. The probe tip contact point can be mathematically described as a hemisphere, which allows for analytical calculation of the strain gradient using the Hertzian model and Boussinesq's calculation.^[^ ^215^ ^]^ The longitudinal or transverse flexoelectric coefficient can be calculated using (Equation 3).

This technique has been used to characterize the flexoelectric properties of single crystals of STO and rutile TiO_2_.^[^ ^15^ ^]^ The experimental setup was also used with monochromatic light to examine the photoflexoelectric effect. A laser with a wavelength of 405 nm was used as the light source, and the illuminated area was around the contact point and at the side of the examined material. The obtained flexophotovoltaic effect is presented in Figure 9C–F.

### 3.5 Compression of a Cone‐Shaped Material

Another procedure utilizes compression of truncated cone‐shaped bulk material (Figure 13C). The truncated cone shape allows the production of a strain gradient in the sample, which is necessary to achieve flexoelectricity. In the experiment, the oscillating force is applied at the top of the sample, and an AC sinusoidal signal controls the compressing frequency. The procedure was adopted for bulk polyvinylidene fluoride (PVDF) with a signal frequency range of 0.5–2.5 Hz.^[^ ^205^ ^]^ The obtained flexoelectric stress gradient constant was 4.55 *×* 10^−6^ pC m N^−1^. The results confirmed the calculations of the flexoelectric strain gradient constant, which was estimated to be ≈0.016 *×* 10^−6^ C m^−1^. The generated electrical charge is measured by the top electrode and detected by the charge amplifier. Based on the collected charge, the transverse flexoelectric coefficient can be determined by calculating the charge on the top surface as follows (Equation 7):

(7) $$Q=\left[\alpha_{2}d_{33}^{ℜ}+\frac{2\left(\mu_{11}-2v\mu_{12}\right)}{Eh}\left(1-\frac{r}{R}\right)\right]F$$

In the equation, *Q* is the collected electrical charge, µ is the transverse flexoelectric coefficient, 𝛼 is the calibration factor, $d_{33}^{ℜ}$ is the measured effective piezoelectric factor, *E* is the Young modulus of the material, *v* is the Poisson ratio, *h* is the height of the sample, *R* is the diameter of the top surface, *r* is the diameter of the bottom surface, and *F* is the compression force. It is important to note that the equation was optimized for the truncated cone‐shaped sample.

### 3.6 Piezoelectric Actuator

An alternative approach for measuring the flexoelectric effect is using a piezoelectric actuator. The piezo‐element delivers oscillatory force to a free end of the cantilever. As a result, the cantilever bends oscillatory and induces alternating polarization between electrodes and electric current flow (Figure 13D). The current is measured using a lock‐in amplifier connected to the electrodes. The strain gradient, which appears during the bending of the cantilever, is derived from deflection amplitude. Mathematically, the effective flexoelectric coefficient (µ_eff_) of a bent cantilever can be described by the following equation:

(8) $$\mu_{\mathrm{eff}}=\frac{\partial \sigma }{\partial G}=\frac{\frac{\partial \left(nd\right)}{\partial \phi }*\partial \phi }{\partial G}=\sqrt{\frac{{n_{0}}_{r}}{2\phi_{0}}}\;*\varphi \frac{t}{2}$$

where σ is the free carrier density at the metallic surface (σ = *nd*), where *n* is the density of free carriers, and *d* is the depletion width; *G* is the curvature (strain gradient), φ is the surface strain, 𝜀_0_ is the dielectric permittivity, 𝜀_r_ is the relative dielectric constant, *t* is the crystal thickness, and *ϕ*_0_ is the Schottky barrier height (given in eV).^[^ ^88^ ^]^

## 4 Methods of Indirect Study

A further strategy for studying the flexoelectric effect is founded on indirect analysis. The flexoelectric coefficient can be extracted indirectly from various physical phenomena closely related to flexoelectricity, but the analysis approach differs from current or electric polarization measurements.^[^ ^73^, ^213^ ^]^

### 4.1 Piezoresponse Force Microscopy (PFM)

One indirect method for studying flexoelectric properties is piezoresponse force microscopy (PFM), a surface characterization method based on scanning probe microscopy (SPM). In this technique, the surface of a sample is scanned with a conductive AFM probe and voltage applied to it. The local electric field leads to the piezoelectric response that causes the studied material to deform. The AFM probe detects the expansion or contraction of the sample by measuring the surface topography.^[^ ^216^ ^]^

The PFM technique can map the spatial distribution of piezoelectricity and ferroelectricity in materials and study their local domain structure. However, since every dielectric, even nonpiezoelectrics, gives an electromechanical response in PFM, determining whether the sample is genuinely piezoelectric is intensely demanding. Therefore, it is crucial to understand the trustworthy source of the induced polarization or deformation in the sample. It was shown that inverse flexoelectricity can produce a significant PFM signal in nonpiezoelectric materials such as STO.^[^ ^217^ ^]^ This makes the method suitable for the characterization of inverse flexoelectricity, where the flexoelectric coefficient (µ*_ijkl_*) can be extracted with the following equation (9):

(9) $$\sigma_{ij}=\mu_{ijkl}\frac{\partial E_{k}}{\partial x_{l}}$$

where 𝜎*_ij_* is the stress tensor and *E_k_* is the electric field^[^ ^197^ ^]^ (Figure 13E).

The PFM method allows for qualitatively distinguishing the piezoelectric and flexoelectric contributions in the sample. In the case of flexoelectricity, the generated signal depends on the probe tip contact radius, while it is constant for the piezoelectric effect. The probe tip contact radius changes with the applied contact force.

### 4.2 Nanocompression Testing

Nanocompression testing allows for the determination of the longitudinal flexoelectric coefficient. This method studies the change in the stiffness of dielectric material caused by external mechanical force. It has been observed that the flexoelectric effect causes a change in stiffness, which is particularly significant at a small scale.^[^ ^218^ ^]^ Therefore, an analytical model was proposed in which the nanocompression force and nanocompression displacement were studied for column‐ and cone‐frustum‐shaped samples. The experimental setup is shown in Figure 13F. Figure 13F(a,b) shows the two samples of different shapes. The stress caused by the nanocompression is homogenous in the column‐shaped sample and inhomogenous (resulting in a stress gradient) in the cone‐frustum‐shaped sample, as shown in Figure 13F(c,d). The stress gradient causes polarization due to the flexoelectric effect, which induces a reduction in the stiffness of the material. This effect is significantly larger in smaller samples or for higher half‐cone angles. For the constant cross‐section pillar, this effect is not present; therefore, measuring the changes in stiffness between the two shaped samples allows for the determination of the flexoelectric coupling coefficient. The advantage of the above method is that it does not require any measurement of electronic polarization or current output, as it only requires the mechanical measurement of the stiffness reduction.

### 4.3 Second Harmonic Generation (SHG) Analysis

The second harmonic generation (SGH) analysis method is a noncontact characterization technique based on generating the second harmonic, an optical phenomenon in which a photon of a specific frequency interacts with a nonlinear medium to generate a new photon with twice the original frequency. This method allows for a thorough indirect investigation of the flexoelectric effect by examining the strain gradients. From SHG analysis, one can provide information on symmetry breaking in the material since this effect does not appear in centrosymmetric structures. It also includes information regarding deformation patterns and crystal symmetry.

Raman spectroscopy further supplements the analysis, as the Raman shift marks the stressed regions where strain gradients are induced. Combining these two methods makes it possible to observe the loss of the inversion center upon inducing the strain gradient, indicating a flexoelectric effect.

This method was used to study the 850 nm deep indentation pattern imprinted on single‐crystal STO. SHG allowed for the observation of noncentrosymmetric structures near the inhomogeneously strained regions. These results suggested the appearance of flexoelectricity in STO single‐crystal deformed by nanoindentation. The topography of the structures is shown in Figure 12D,E. The AFM‐obtained images confirmed that there are no cracks on the surface of the crystal due to the process of nanoindentation and that the process generated just elastic deformations. Additionally, Figure 12B,C,F presents the dependency of the flexoelectric coupling effect, dielectric susceptibility dependence, and associated with the indentation process load–displacement curve, respectively.

### 4.4 Laser Doppler Vibrometer (LDV)

A Rrecently published paper, where LDV on amorphous HfO_2_ was tested, proposed a novel method based on laser light, which is pointing at the cantilever beam to calculate its displacement. Experimental setup additionally requires a lock‐in amplifier to eliminate the noise signals and read the actual data coming from the LDV. Moreover, the lock‐in amplifier keeps track of the resonance frequency with the Phase Locked Loop. Calibration is done using the thermomechanical noise, which involves recording the noise at the resonance frequency, which is later compared to the theoretically expected value.

To calculate the effective flexoelectric coefficient (𝜇_𝑒ff_), the following equation is used:

(10) $$\mu_{\mathrm{eff}}=\frac{V_{LDV}}{0.445*V_{\mathrm{actuation}}*\;L^{2}Q}\;*\sqrt{\frac{4Qk_{B}T}{\frac{1}{4}L*W*\sum \rho_{i}t_{i}*\omega_{0}^{3}*PSD}}*D_{f}$$

where *V*_LDV_ is the measured voltage in the lock‐in amplifier, *V*_actuation_ is the actuation voltage, *L* is the length of cantilever, *Q* is the quality factor, *k*_B_ is the Boltzmann constant, *T* is the temperature, *W* is the width of cantilever, 𝜌*_i_* is the density of cantilever's material, *t* is the thickness of cantilever, *ω*_0_ is the resonance frequency, PSD stands for power spectral density (in 𝑉^2^/𝐻z), and D*_f_* is the flexural ridgity (which have to be calculated for selected material).

The described method is only limited by the thermomechanical noises, which means that minimal cantilever movement by flexoelectric effect has to be at the same level as the mentioned noises. Speaking of the numbers, the detection limit calculated by the Authors is 1 × 10^−15^ C m^−1^. More details about the method can be found in the reference.^[^ ^140^ ^]^

## 5 Clean Energy Generation

This review has covered the fundamental principles of flexoelectricity, including its origins and how material properties like shape, size, and applied stress influence it. We have also discussed theoretical and experimental modeling approaches. A key focus of this review has been the various methods for measuring flexoelectricity, both directly and indirectly, using cutting‐edge techniques. This accurate measurement is crucial for understanding the behavior and applicability of these materials and their effect on diverse catalyst applications.Photocatalysis has been explored to generate solar fuel,^[^ ^219^ ^]^ dye degradation,^[^ ^220^ ^]^ and other environmental applications.^[^ ^221^ ^]^ Physically, when a photocatalyst is irradiated by sunlight, it becomes excited, which causes the creation of valence‐band holes and conduction‐band electrons (as shown in the equation below). Holes are known to exhibit oxidation, while electrons are known to show a reduction capability. In recent years, photocatalysts based on already mentioned SrTiO_3_ (STO),^[^ ^222^ ^]^ and TiO_2_,^[^ ^141^ ^]^ have a well‐established photocatalytic response, mainly in ultraviolet light, have been finding more applications when combined in nanocomposites, which, apart from the visible light response, also have higher charge mobility and better separation efficiency, due to the heterojunction formation.^[^ ^223^, ^224^, ^225^, ^226^, ^227^, ^228^ ^]^

As mentioned in the introduction, the presence of strain gradients invokes the flexoelectric effect. Notably, this affects catalytic materials with electric polarization (as shown in the Equation 11). It will depend largely on the choice of material, which may cause other concomitant effects (such as piezoelectric, ferroelectric, etc.), resulting in modulation of the dynamics of interfacial charge carriers. Consequently, this modulation affects the energetic profiles of active sites, reaction intermediates, and the energies required for reactant adsorption/product desorption, creating alternative reaction pathways and affecting the efficiency of catalytic transformation, which was recently confirmed by theoretical calculation and experiment (tabulated in **Table** **2**). Besides this, catalyst morphology, local defect sites, cracking edge‐site defects, and structural phase significantly affect the flexoelectric generation.^[^ ^229^ ^]^ The following reaction occurs during the flexoelectric and photoenhanced flexoelectric reactions.

Flexoelectricity:

(11) $$\mathrm{External}\;\mathrm{stimuli}=\mathrm{Net}\;\mathrm{Polarization}=h^{+}+e^{-}$$

Photo‐enhanced flexoelectricity:

(12) $$\mathrm{External}\;\mathrm{stimuli}+\mathrm{light}\;\mathrm{irradation}\;=\;\mathrm{Net}\;\mathrm{Polarization}\;=h^{+}+e^{-}$$

Interaction with reactant:

(13) $$H^{+}+\;\;e^{-}=H^{•}$$

(14) $$H^{•}+\;\;H^{•}=H_{2}$$

(15) $$OH^{-}+h^{+}=OH^{•}$$

(16) $$O_{2}+\;\;e^{-}={\;O}_{2}^{-}$$

(17) $$H_{2}O\;+h^{+}=H^{+}+\;OH^{•}$$

(18) $$\mathrm{Dye}+O_{2}^{-}/OH^{•}=\mathrm{degradation}\;\mathrm{product}$$

**Table 2:** Compiles the recent literature utilizing flexocatalysis as a driven force for catalytic applications.

Material	Reaction condition	Application	Refs.
Hydroxyapatite@fluorapatite core–shell nanorods (HAP@FAP)	Reactant loading 200 mg kg^−1^, catalyst = 20 mg (1%), *V*_w_/*M*_s_ = 20 : 2 (mL g^−1^), under ultrasonication	Phenanthrene (PHE) degradation in soil	[260]
Ag(CuZn)(AlCr)_2_O_4_ and CuO	Under ultrasonication	H_2_ evolution reaction (HER)	[259]
MnO_2_ nanoflowers	5 mg catalyst in ultrasonication with irradiation (320 W, 40 kHz)	Methylene Blue dye degradation	[9]
Single‐atom Pt‐loaded graphitic carbon nitride	Under ultrasonication 10 mg catalyst in RB (10 ppm) For HER: 2.5 mL of (methanol), 7.5 mL of distilled water, and 10 mg catalyst	Rhodamine B (RB) dye degradation, H_2_ evolution	[245]
δ‐MnO_2_ and TiO_2_	5 mg catalyst 50 ml (10 ppm MB) under the presence of light 365 nm and ultrasonication	Methylene Blue (MB) dye degradation	[105]
BaTi_2_O_5_	Ultrasonication (200 W, 40 KHz). For HER: 5 mg catalyst 10 ml DI water with 0.05 Na2SO3 as sacrificial agent under sonication. 2 mg catalysts in 10 ml dye solution (5 mg/L)	H_2_ evolution reaction (HER) and dye degradation (RhB)	[112]
Mo@(2H‐1T)‐MoSe_2_	1 cm × 2 cm catalyst submerged into 50 mL of dye solution(10 ppm) with ultrasonication (40 KHz)	RhB degradation	[229]

Such oxygen radicals are crucial for dye degradation, while a light‐enhanced flexoelectric technique called the photoflexoelectric effect may increase the rates of solar‐to‐fuel conversion. This effect offers a perspective on catalytic processes and is characterized by electrical polarization and light‐induced strain gradients.^[^ ^230^ ^]^ Flexoelectric materials show promise for catalytic innovation because of their strain gradient‐induced surface electrical polarization. Strain gradients are caused by pressure and concentration changes that ubiquitously occur on catalyst surfaces during chemical reactions at an industrial scale. Therefore, flexoelectric‐based catalytic strategies present a promising opportunity to advance catalytic processes.^[^ ^231^ ^]^ However, such flexoelectric‐based catalytic studies are scarce. In the following section, we will discuss the role of flexoelectric materials in clean energy production and catalysis, most of which are present in nanocomposite forms and show functional heterojunctions with active flexoelectric materials.

### 5.1 Flexoelectric Materials in Clean Energy Production or Conversion

The crosslink between flexoelectricity and catalysis is a novel area of research and has the potential to create more efficient and effective catalysts. One of their uses is clean energy production or conversion. For example, Mahele et al. synthesized an atomically thin layer of niobium carbide (NbCx) by carburization of the niobium salt NbCl_5._ It has sufficient mechanical strength, chemical stability, and electrical conductivity to function as nanogenerators for power generation. Their research findings showed that repeated mechanical actions like rubbing, pushing, pulling, and moving can produce flexoelectricity. In addition, they have a number of advantages over conventional batteries, such as small size, high power output, flexibility, environmental friendliness, and durability.^[^ ^213^ ^]^ However, the observed flexoelectricity is most likely the result of the strong piezoelectric response of oxygen‐functionalized MXenes.^[^ ^232^ ^]^

**Figure 14:**
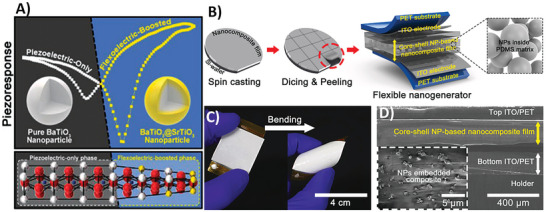
Schematic illustration A) piezoelectric response in pure BaTiO_3_ and core–shell BaTiO_3_ @SrTiO_3_. B) Nanocomposites sandwiched between the two ITO‐coated plastic substrates to characterize piezoelectric output performance. The right panel of C) showing the fabricated sample bent by human fingers confirms that nanocomposite is sufficiently mechanically stable and flexible during repeatedly bending motions. D) The cross‐sectional SEM images of the nanocomposite layer between the top and bottom plastics (the inset: a magnified image). Image taken with permission from reference.^[^ ^50^ ^]^ Copyright 2021 Elsevier.

Kim et al. fabricated BTO‐STO core–shell structures (**Figure** **14**A) using a simple hydrothermal reaction with a well‐controlled shell thickness, and the shell thickness was optimized by changing the hydrothermal reaction time and then incorporating two indium tin oxide (ITO)‐coated plastic substrates to characterize the piezoelectric output performance (Figure 14B). The experimental investigation showed that the effective piezoelectric charge coefficient increased by nearly 200% due to the combined effect of flexoelectricity, as shown in Figure 14A. The fabricated sample bent by human fingers confirmed that the nanocomposite was sufficiently mechanically stable and flexible during repeated bending motions (Figure 14C). The corresponding cross‐sectional SEM images of the top and bottom plastics are shown in Figure 14D. They observed that evenly distributed thicker STO shells around the BTO core component can lessen the associated flexoelectric effect and strain gradient.^[^ ^50^ ^]^

**Figure 15:**
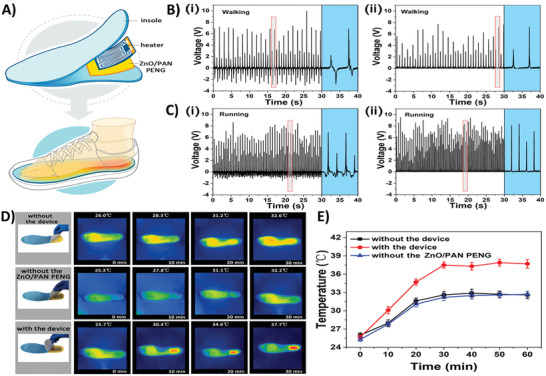
Commercial electric heating sheet to drive a PENG‐generated electric motor. A) A diagram illustrating the self‐heating insole. B) The output voltage produced while walking by the ZnO/PAN‐based nanofabric PENG: i) raw data and ii) rectified data. C) The output voltage produced while operating by the ZnO/PAN‐based nanofabric PENG. D) An IR image of the insole and a digital photo. E) The relationship between the surface temperatures of three insoles with various movement rates. Image adapted with permission from reference.^[^ ^52^ ^]^ Copyright 2020, American Chemical Society.

In the same line, Sun et al. fabricated the ZnO/PAN nanofabrics by integrating zinc oxide nanorods with polyacrylonitrile. It enhanced pressure sensitivity and vibrational energy harvesting ability by about 2.7 times that of a pristine PAN nanofiber, and a maximum output power density of 10.8 mW m^−2^ was obtained. It even functions as a reliable sensor to track human activity (walking and running) as shown in **Figure** **15**A–F. Apart from that, when piezoelectric nanofabric is connected to a sheet heater for personal thermal management, such as winter foot.

The flexoelectric effect has emerged as a focal point in the field of nanogenerators (NGs), offering promising prospects for efficiently converting mechanical vibrations into electrical energy. For instance, Qi et al.^[^ ^233^ ^]^ observed enhanced piezoelectric properties in wavy PZT ribbons attributed to the flexoelectric effect. Their methodology involved stretching polydimethylsiloxane (PDMS) and attaching it to a magnesium oxide substrate, facilitating the transfer of 500 nm thick PZT ribbons onto the PDMS. Upon releasing the prestrained PDMS, the PZT ribbons adopted sinusoidal waveforms with dimensions of 80 µm wavelength and 11 µm height. This arrangement induced a midplane uniaxial strain of 8.5 MPa and a strain gradient of 3 × 10^4^ m^−1^, surpassing those achievable through compression or bending tests on bulk materials. The substantial strain gradient led to notable charge separation, augmenting the piezoelectric effect by up to 70% compared to flat portions of PZT ribbons without flexoelectric contribution. In another study by Wang et al.,^[^ ^234^ ^]^ a bent PZT diaphragm exhibited a large strain gradient (≈10^2^ m^−1^), resulting in a high flexoelectric polarization of ≈1 µC cm^−2^. This heightened flexoelectric polarization improved diaphragm performance and influenced the hysteresis loop. Furthermore, the flexoelectric coefficient of these PZT thin films was significantly higher (2 × 10^2^ µC m^−1^) compared to bulk PZT material. Additional contributions include the development of an analytical model for a nanoscale energy harvester based on the flexoelectric effect by Wang and colleagues,^[^ ^235^ ^]^ and Choi et al.^[^ ^210^ ^]^ reported Zn–Al:LDHs capable of converting mechanical deformation into electrical output through flexoelectricity. These advancements underscore the potential of the flexoelectric effect in enhancing the performance and versatility of nanogenerators across various applications.

In addition to flexoelectric nanogenerators, flexoelectric sensors are emerging as notable devices in various domains, owing to their compact dimensions, minimal susceptibility to aging, and low risk of. These sensors find applications in crucial areas such as crack detection, structural health monitoring, and curvature detection. Yan et al.^[^ ^236^ ^]^ pioneered the development of a flexoelectric sensor based on barium strontium titanate (BST), designed to convert bending deflections into electrical signals. Their approach involved symmetrically attaching two microcurvature BST sensors to the center of an aluminum beam, enabling the detection of deflections. The resulting charge output exhibited direct proportionality to the strain gradient. Merupo et al.^[^ ^237^ ^]^ utilized polyurethane films on a bilayered substrate composed of polyethylene terephthalate (PET) and aluminum foil to detect curvature induced by bending motion. Meanwhile, Huang et al.^[^ ^238^ ^]^ introduced a flexoelectric strain gradient sensor based on Ba_0.64_Sr_0.36_TiO_3_ for structural health monitoring and crack detection, boasting a transverse flexoelectric coefficient of 45 µC m^−1^. Kwon et al.^[^ ^239^ ^]^ engineered a multilayered flexoelectric sensor utilizing BST ceramic, achieving a nearly threefold increase in charge output compared to single‐layer configurations. Finally, Hu et al.^[^ ^240^ ^]^ developed a distributed sensing system employing BST ceramic bars on a flexoelectric cantilever beam, showcasing effective detection of distributed strain gradients. This distributed sensing paradigm holds significant promise for applications in structural monitoring and the design of intelligent structures.

The converse flexoelectric effect holds significant promise in the field of actuators, showcasing the potential for substantial improvements in their performance. Within microelectromechanical and nanoelectromechanical systems (MEMS and NEMS), the cantilever serves as a fundamental component, essentially acting as a self‐supporting capacitor. Efforts have been directed toward characterizing the converse flexoelectric coefficients using diverse materials, such as barium strontium titanate (BST), employing trapezoidal block samples. Such investigations have unveiled flexoelectric displacements at angstrom levels.^[^ ^241^ ^]^ Similarly, Shen et al. examined the converse flexoelectric effect in a PZT microbeam outfitted with interdigitated electrodes.^[^ ^242^ ^]^ Under 3 V excitation, deflections attributed to flexoelectric and piezoelectric effects were measured at 7.25 and 6.14 nm, respectively. While the flexoelectric effect is typically weaker than the piezoelectric effect in bulk materials, its significance is amplified in MEMS due to the slender nature of MEMS structures and the inverse correlation between strain gradients and thickness. Notably, Bhaskar et al.^[^ ^243^ ^]^ demonstrated that the flexoelectric effect can either amplify or dampen the piezoelectric response of a cantilever, contingent upon the ferroelectric polarity. This phenomenon causes a two‐state asymmetric electromechanical response, hinting at the potential for flexoelectric‐driven actuation through strategic adjustments in bimorph geometry. These insights illuminate the intricate interplay between flexoelectric and piezoelectric phenomena, opening avenues for refining flexoelectric‐driven actuators.

**Figure 16:**
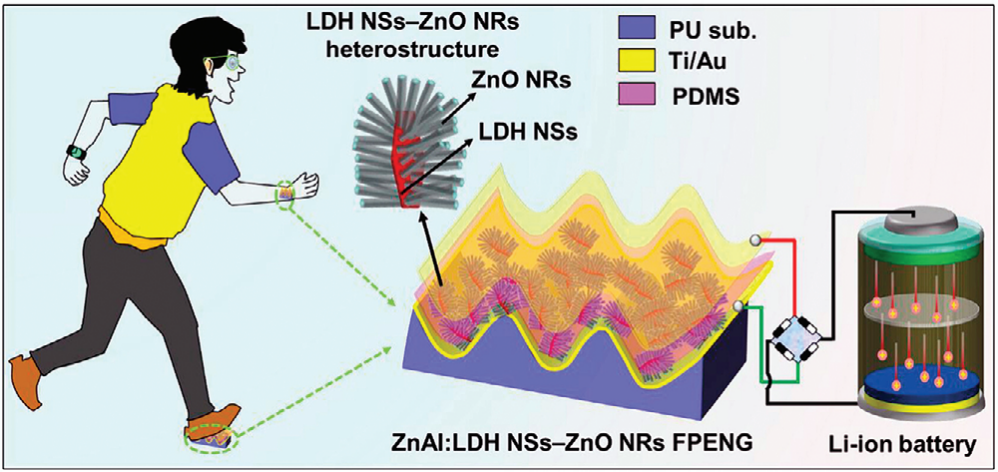
An environmentally friendly, stretchable FPENG with ZnAL:LDH NSs–ZnO NRs is schematically demonstrated for biomechanical energy harvesting and self‐charging Li‐ion battery systems. Image taken with from reference.^[^ ^165^ ^]^ Copyright *2023*, American Chemical Society.

On a similar note, Yoon et al. has fabricated a stretchable flexoelectric–piezoelectric nanogenerator (FPENG) composed of a zinc–aluminum layered double hydroxide nanosheets–ZnO nanorods (ZnAl:LDH NSs–ZnO NRs) heterostructure.^[^ ^165^ ^]^ As shown in **Figure** **16**. Under bending and stretching modes for 4 h showed the best performance with an open circuit voltage of 41.5 V with a current density of 4.57 µA cm^−2^ and a maximum power density of 68.2 µW cm^−2^. This was due to the combined flexoelectric and piezoelectric effects of ZnAl:LDH nanosheet and ZnO nanorods. The same FPENG also performed well under bending and stretching modes for self‐charging a Li‐ion battery by FPENG output under mechanical tapping. Even a commercial calculator was operated by the energy stored in a capacitor.

Thus, materials combining the flexoelectric effect offer a promising solution for energy harvesting and self‐powered systems in wearable electronics and biomedical devices. Eventually, it will be possible to broaden energy production or conversion possibilities. Further, the flexoelectric effect can contribute to photoelectric conversion, which is analogous to the bulk photovoltaic effect,^[^ ^104^ ^]^ where the lack of structural symmetry leads to the asymmetric distribution of photoexcited nonequilibrium carriers in momentum space. One has to remember the flexoelectric effect introduces a symmetry‐breaking perturbation in the structure, enabling the generation of photoexcited nonequilibrium carriers in centrosymmetric materials, such as single crystals of STO, Si, or TiO_2_. The flexophotovoltaic effect is the term used to describe the bulk photovoltaic effect induced specifically by the flexoelectric effect.^[^ ^38^ ^]^ Reports in such fields are scarce and provide new opportunities to integrate light‐induced photovoltaic cells, which might improve the overall clean energy harvesting properties. In another work, the flexoelectric enhancement of the photovoltaic effect was examined in composites of NaNbO_3_, where the flexoelectric coefficient of the NaNbO_3_ nanotube/epoxy composite film was estimated to be 2.77 *×* 10^−8^ C m^−1^, which was about five times larger than for pure epoxy film. An enhancement of photovoltaic current from about 40 to 70 nA cm^−1^ was also noticed.^[^ ^244^ ^]^

### 5.2 Flexoelectric Materials for Catalytic Applications

By exploiting the flexoelectric properties of materials, it may be possible to create catalysts that can be more easily activated or that have enhanced activity due to the electric field generated by a strain gradient. This approach has been explored in several recent studies, which have demonstrated the potential of flexoelectricity to enhance the activity of various catalysts. For example, Wang. et al a graphitic carbon nitride (SA‐Pt/CN) loaded with a single atom of Pt, and evaluated its piezo–flexo catalytic capabilities by performing a Rhodamine B (RB) dye degradation test and hydrogen evolution reaction (HER) in the absence of light using ultrasonic vibration.^[^ ^245^ ^]^ The hydrogen gas output of SA‐Pt/CN is 23.3 times higher than that of pristine g‐C_3_N_4_ at 1283.8 µmol g^−1^ h^−1^. Additionally, when compared to the pristine sample, SA‐Pt/CN increases the dye degradation reaction rate by about 2.3 times. The single atom Pt at the N sites of g‐C_3_N_4_ causes lattice distortion and strain gradient expansion in SA‐Pt/CN, disrupting the symmetric structure and enhancing piezoelectric and flexoelectric polarization.

**Figure 17:**
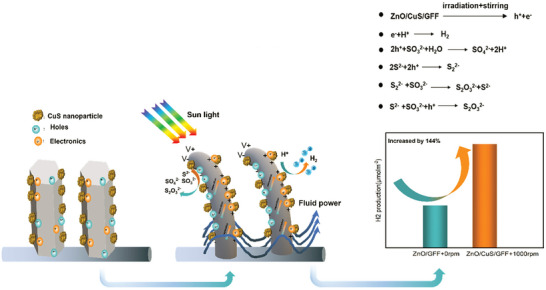
Schematic diagram of hydrogen production by ZnO/CuS/GFF under fluid power and light. Image adapted with permission from the reference.^[^ ^246^ ^]^ Copyright *2023*, American Chemical Society.

Similarly, as illustrated in **Figure** **17**, J. He et al. synthesized free‐floating ZnO/CuS/GFF by loading ZnO/CuS nanorod arrays onto flexible glass fiber fabric (GFF).^[^ ^246^ ^]^ The ZnO nanorods generate a piezoelectric field in response to gentle mechanical stimulation from fluid power, facilitating the efficient separation of photogenerated carriers. It results in an impressive 53.45% increase in the hydrogen production rate, which reaches the value of 3479.52 µmol m^−2^ h^−1^. The efficiency even surpasses that of piezo‐photocatalysts stimulated by light and ultrasonic waves. As known, photocatalytic reactions have major limitations in the recombination events occurring at the nanosecond scale. An efficient separation is necessary at the catalyst surface to undergo the chemical reaction. However, flexoelectric material‐modified photocatalysts enhance the electron–hole rearrangement due to lattice distortion and strain gradient expansion. It increases the reactant interaction with the photocatalysts' surface. For example, flexophotocatalytic degradation of methyl orange (MO)/rhodamine B (RhB)/MB mixture dyes and other organic pollutants, including tetracycline hydrochloride (TC) and ciprofloxacin (CIP) were investigated using Ag_2_MoO_4_ particles.

**Figure 18:**
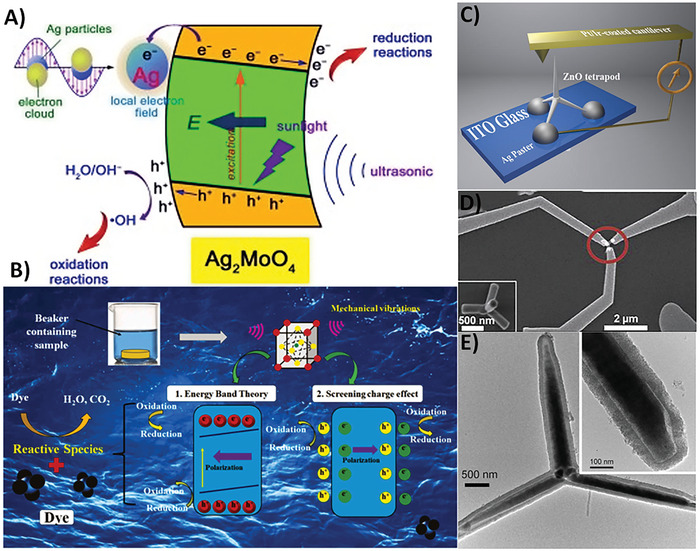
A) Schematic illustration of photogenerated electron–hole pairs in Ag_2_MoO_4_, which is separated by flexoelectricity, leading to an increased photocatalytic process. Image taken with permission from the reference.^[^ ^36^ ^]^ Copyright 2021, Elsevier. B) Plausible mechanism of degradation of MB dye via piezocatalysis process by proposed energy band gap and screening charge theory. Image taken with permission from reference.^[^ ^247^ ^]^ Copyright 2024, Elsevier. C) Electronic sensing device, combining UV light and force, based on ZnO – schematicly. Image taken with permission from the reference.^[^ ^248^ ^]^ Copyright 2013, AIP Publishing. D) ZnO p‐n junction diode–tetrapod‐based electronic device, inset shows ZnO tetrapod before lithographic process. Image taken with permission from the reference.^[^ ^249^ ^]^ Copyright 2009, AIP Publishing. E) Transmission electron microscope imaging of TiO_2_‐coated (light gray) ZnO tetrapods (dark gray, black) used as highly effective photocatalysts. Image taken with permission from reference.^[^ ^250^ ^]^ Copyright 2007, Elsevier.

During photocatalysis, the simulated sunlight generated electrons in their CB and holes in their VB. The photogenerated carriers interact with organic contaminants in a reductive or oxidative manner, either directly or indirectly, resulting in the photocatalytic breakdown of organic pollutants. If no further action is applied to the catalyst, the photogenerated electrons and holes are recombined; nevertheless, the limited utilization of the photogenerated carriers restricts the catalyst's photocatalytic activity. When ultrasonic irradiation is used with photocatalysis, the Ag_2_MoO_4_ crystals are vibrated, causing a mechanical strain gradient and thereby inducing polarization or flexoelectricity. As a result, the mechanical strain gradient caused the polarization field to move the CB electrons and VB holes in opposite directions, resulting in the effective separation of the photogenerated carriers, as shown schematically in **Figure** **18**A. Hence, more photogenerated carriers are accessible to participate in redox processes, resulting in a considerable increase in the catalytic degradation efficiency of organic contaminants.^[^ ^36^ ^]^ Ag_2_MoO_4_ is another well‐studied material for photocatalysis^[^ ^251^, ^252^, ^253^ ^]^ with a cubic structure commonly known as β‐Ag_2_MoO_4_. It is also very well known for its photodegradation properties^[^ ^254^ ^]^ with a wide bandgap of about 2.5–3 eV,^[^ ^251^, ^255^ ^]^ which causes its excitation only under ultraviolet light. This can be changed to visible light ranges by partially substituting Cr for Mo.^[^ ^256^ ^]^ Furthermore, several investigations reveal that the photocatalytic activity of Ag_2_MoO_4_ is significantly reliant on its shape and synthesis process. Some research has shown that replacing Mo with Cr improves photocatalytic activity by more than thrice.^[^ ^36^ ^]^ Interestingly, the synergistic effect of flexoelectricity and morphology were investigated for MnO_2_ in the form of nanoflowers for pollution degradation processing. After exposure to the pollutant, MnO_2_ nanoflowers were put at different ultrasonic powers under constant room temperature.^[^ ^257^ ^]^

Regarding the screening charge effect, these mechanisms are distinguished by the fact that the charges actively involved in these reactions are surface‐adsorbed screening charges that originate from an external system, as opposed to internal charges originating from the material itself. Figure 18B illustrates the likely mechanism of the piezocatalysis process using screening charge theory in addition to energy band gap theory.

**Figure 19:**
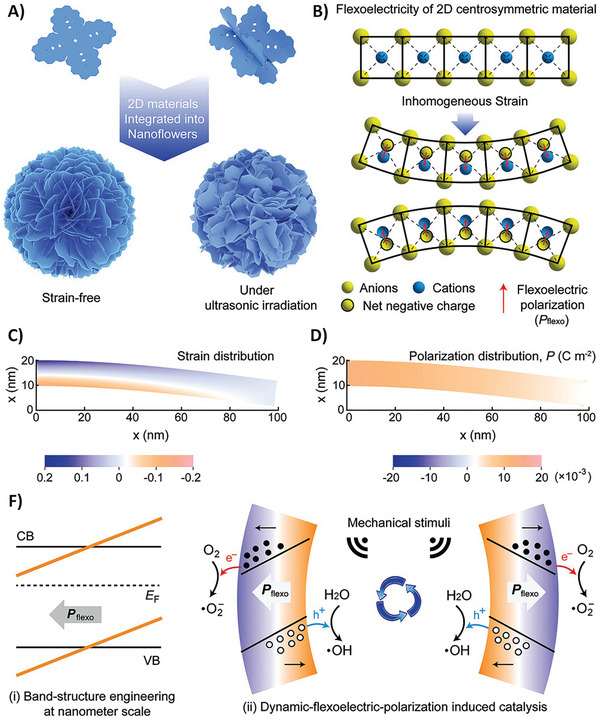
Schematic illustration of flexocatalysis mechanism in 2D centrosymmetric semiconductor. A) A nanoflower‐like structure composed of 2D nanosheets is exposed to ultrasonic irradiation in the absence of strain. Each nanosheet behaves like a cantilever beam and undergoes mechanical deformation due to the vibration. B) An inhomogeneous strain applied to the nanosheet causes a relative displacement between the cationic and anionic centers, generating a flexoelectric polarization across the nanosheet. C,D) A cantilever beam model is used to simulate the flexoelectricity in 2D materials, showing the strain distribution (C) and the flexoelectric polarization distribution (D) under a 100 nN force. E) The proposed flexocatalysis mechanism for organic pollutants degradation involves: i) the creation of an internal flexoelectric polarization that modifies the band structure of the 2D semiconductor. CB, VB, and EF denote the conduction band, valence band, and Fermi level, respectively. The black and orange lines indicate the band structures of the 2D semiconductor without and with strain‐gradient, respectively; ii) the separation and transport of electrons and holes and the formation of reactive species that catalyze the degradation of organic pollutant. Image taken with permission from the reference,^[^ ^9^ ^]^ Copyright 2022, Wiley.

The schematic diagram (**Figure** **19**A) demonstrates the flexocatalysis mechanism in a two‐dimensional centrosymmetric semiconductor, a nanoflower‐like 2D nanosheets of MnO_2_. When exposed to ultrasonic irradiation, this structure undergoes mechanical deformation, as illustrated in Figure 19B. This deformation is caused by an inhomogeneous strain applied to the nanosheet, resulting in a flexoelectric polarization across the nanosheet. Figure 19C,D displays the strain distribution and flexoelectric polarization distribution, respectively, which were simulated using a cantilever beam model. However, cyclic experiments reveal a declining degradation rate, suggesting damage to the nanoflower structure. Subsequent investigations involving MnO_2_ nanoflowers emphasize the necessity of catalysis for pollutant degradation. The proposed flexocatalysis mechanism for organic pollutant degradation, detailed in Figure 19E, involves the creation of an internal flexoelectric polarization that modifies the band structure of the 2D semiconductor, facilitating the separation and transport of electrons and holes. The structural aspect, particularly the flower‐like architecture, emerges as crucial due to its flexoelectric response. Notably, almost complete pollutant degradation is achieved after 30 min of each cycle, highlighting the proposed approach's effectiveness in environmental remediation.^[^ ^105^ ^]^

Apart from the experimental studies, comprehensive first‐principles calculations have also validated that flexoelectricity has been accountable for the bandgap alignment arrangement of transition metal dichalcogenide (TMDC) heterostructures.^[^ ^258^ ^]^ The combined effect of curvature‐induced flexoelectricity and circumferential tensile strain causes a rapid lowering of the conduction band minimum as the diameter of a TMDC nanotube decreases. In contrast, the valence band maximum exhibited an initial decrease before increasing.

**Figure 20:**
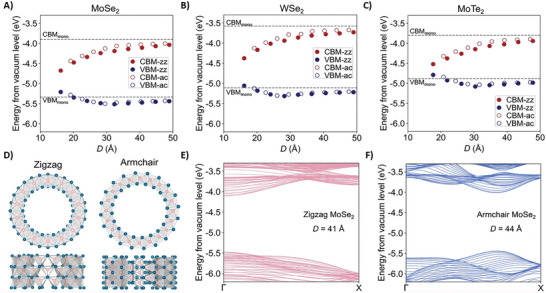
MoSe_2_, WSe_2_, and MoTe_2_ nanotube band‐edge levels as functions of diameter. The VBM and CBM of MoSe_2_, WSe_2_, and MoTe_2_ nanotubes evolve with tube diameter in the following ways: (A–C). D) Atomistic depictions of transition metal dichalcogenide (TMDCs) armchair and zigzag nanotubes seen from the side (bottom panels) and along the tube axis (top panels). The spheres in cyan and pink represent the spheres. Atoms of chalcogens and transition metals. E,F) The electronic band structures of armchair (F) and zigzag (E) nanotubes have diameters of ≈40 Å. This symmetry causes only half of the 1D Brillouin zone to be visible, with the electron wave vectors running parallel to the tube axis. Image adapted with permission from the reference.^[^ ^258^ ^]^ Copyright 2023, Springer Nature.

Their result findings show that individual TMDC nanotubes form coaxial heterostructures, and in heterostructural systems such as large‐diameter MoSe_2_@WS_2_, MoTe_2_@MoSe_2_, and MoTe_2_@WS_2_, the combined effect of diameter‐dependent band‐edge levels and intertube coupling via flexovoltage can result in a shift in intertube band alignment from Type II to Type I semiconductor. Although the overall pattern of variation in the valance band maxima (VBM) and conduction band maxima (CBM) levels of MoSe_2_, WSe_2_, and MoTe_2_ nanotubes is comparable to that of MoS_2_ and WS_2_, the nonmonotonic shift in their VBM is even more noticeable as depicted in **Figure** **20**A–F. Interestingly, until the tube diameter falls below about 30 Å, the VBM of the three Se‐ and Te‐based TMDC nanotubes does not increase. For MoSe_2_, WSe_2_, and MoTe_2_ nanotubes, the corresponding transition diameters are ≈30, 28, and 30 Å. At what are known as the “transition diameters,” the VBM falls to a maximum of 0.2 eV in relation to the corresponding monolayers.

In their seminal study, Pu et al. utilized first‐principles calculations to explore the impact of in‐plane tensile loadings on the chemical reactivity of monolayer transition metal dichalcogenides (TMDs) MX_2_ (where M = Mo or W, and X = Te, Se, or S) in water splitting and hydrogen evolution, with a focus on grain boundaries (GBs).^[^ ^35^ ^]^ Their investigation revealed a noteworthy augmentation in the chemical activities of monolayer TMDs when GBs were present compared to those lacking GBs. This enhancement arises from the flexoelectric effect induced by nonuniform deformation and strain gradient, resulting in amplified charge polarizations of the X and M atoms at the GB sites. Consequently, this facilitated the dissociation of water molecules on the GB sites and concomitantly reduced the reaction barrier of the hydrogen evolution reaction. Moreover, their findings demonstrate that the energy barriers for water molecule splitting and the hydrogen adsorption‐free energies on the GB sites decrease with increasing flexoelectric effects.

**Figure 21:**
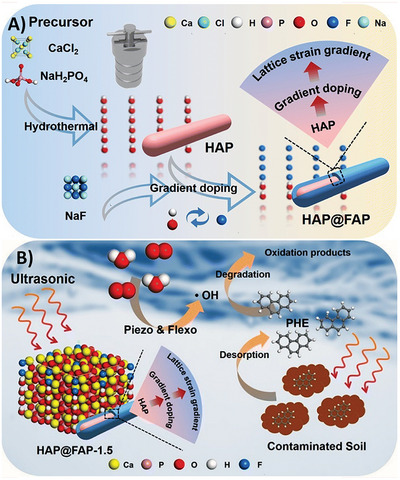
Schematic illustration of the A) fabrication of HAP@FAP. B) Proposed mechanism for piezocatalytic degradation by HAP@FAP. Image taken with permission from the reference,^[^ ^260^ ^]^ Copyright 2023, Royal Society of Chemistry.

Besides the flexoelectric effect in GB‐containing TMDs to enhance their surface catalytic activity for hydrogen generation, Wu et al. synthesized a novel high‐entropy oxide nanocomposite featuring Ag(CuZn)(AlCr)_2_O_4_ and CuO phases, characterized by an intricately hierarchical wrinkled surface.^[^ ^259^ ^]^ Applying a mechanical force induced a nonhomogeneous strain gradient at the interface between the two phases, altering the local charge distribution and generating flexoelectric polarization, effectively prolonging electron/hole recombination. Remarkably, the nanocomposite exhibited a hydrogen production rate of 2116 µmol g^−1^ h^−1^ without external light irradiation, surpassing the hydrogen yield achieved through photocatalysis by 980%. Furthermore, the presence of a strong electrical field at the interface between the Ag(CuZn)(AlCr)_2_O_4_ and CuO phases indicated the establishment of a flexoelectric potential (flexopotential) at the structural boundaries owing to the localized strain gradient. Such findings underscore the potential of nanocomposites. Further, a heterogeneous core–shell structure of HAP@FAP was successfully fabricated through surface‐gradient F‐doping in hydroxyapatite, aimed at enhancing the piezocatalytic degradation of phenanthrene (PHE) in soil breaking down 79% of the PHE (initially at 200 mg kg^−1^ in soil) within 120 min,^[^ ^260^ ^]^ as shown in **Figure** **21**A,B. This exceptional degradation efficiency can be attributed to two main factors. First, flexoelectricity induced by the chemical heterogeneity‐induced lattice strain gradient substantially improved the piezoelectric coefficient, enhancing the piezocatalytic activity. Second, ultrasound application effectively promoted the desorption of PHE from the soil into the aqueous phase, where the oxidation process was most efficient, further boosting the degradation efficiency. Additionally, the HAP@FAP composite has advantages such as good biocompatibility, low cost, and wide availability, making it well‐suited for in situ soil remediation applications.

Similarly, Gaur et al. studied the surface flexoelectricity induced on BaTiO_3_ ceramic pellets to enhance their piezocatalytic efficiency.^[^ ^247^ ^]^ This involved strategically exposing the pellets to 150 °C for 2 h, then rapidly cooling them to room temperature. This temperature treatment created oxygen vacancies within the pellets, resulting in a strained surface and the generation of a flexoelectric response. XPS analysis confirmed the presence of oxygen vacancies, while the nanoindentation technique revealed an induced strain on the ceramic surface. The elevated piezopotential, validated by PFM, significantly enhanced piezocatalytic performance. Quantitatively, the fast‐cooled BaTiO_3_ pellets exhibited ≈73% degradation of MB dye, surpassing the 50% degradation achieved by sintered/polished BaTiO_3_ in a 4‐h piezocatalysis process. This 1.47‐fold enhancement in MB dye degradation was attributed to the flexoelectric response induced by the heat treatment process. Unlike chemical processes, such as doping, the flexoelectric effect arises from physical phenomena, with parameters such as load application during pellet synthesis, temperature optimization post‐sintering, and abrasion depth playing crucial roles. Despite these considerations, the fabrication process associated with flexoelectricity is relatively straightforward compared with specific chemical processes, simplifying the overall synthesis. Therefore, the flexoelectric response of BaTiO_3_ ceramics was additionally investigated for its application in the degradation of dyes. Similarly, Chen et al. reported the exceptional capability of TiO_2_ nanosheets in decomposing dye molecules via flexoelectric field‐driven catalysis in the absence of light irradiation, resulting in a notable hydrogen evolution rate of ≈275.96 µmol g^−1^.^[^ ^261^ ^]^ Their combined theoretical calculations and experimental observations confirmed the significant contributions of strain‐induced flexoelectricity and bandgap reduction to the catalytic reactions on the nanosheets. Specifically, strain gradient‐induced flexoelectricity exhibited a superior enhancement in catalytic activity compared to TiO_2_ nanoparticles. They employed defect engineering to investigate the strain sensitivity, which is crucial for augmenting flexopotential‐driven electrochemical reactions. Piezoresponse force microscopy (PFM) results revealed a decrease in the effective flexoelectric coefficient with increasing tip force, with TiO_2_ nanosheets of 50 nm thickness exhibiting a high flexoelectric coefficient of 39.04 pm V^−1^. Finite element method (FEM) simulations further illustrate the superior flexopotential distribution of TiO_2_ nanosheets over nanoparticles, elucidating how the strain gradient induces a larger flexopotential in nanosheets. In addition, our model elucidates the effects of strain‐induced bandgap reduction and band structure modulation on the catalytic process. Oxygen vacancies enhance catalysis by increasing the water adsorption capacity and facilitating the self‐dissociation of water, thereby enhancing hydrogen evolution efficiency. These findings offer novel insights into flexocatalysis of 2D TiO_2_ nanosheets for environmental remediation and hydrogen production, paving the way for innovative designs of centrosymmetric 2D TiO_2_ nanomaterials and advancing catalytic activity research. It highlights the significant role of surface flexoelectricity in enhancing material piezocatalytic potential and suggests promising avenues for future environmental remediation applications. Moreover, the Table 2 provides an overview of recent literature on flexocatalysis, showcasing its diverse applications in catalysis.

Overall, we have reviewed the current and exciting developments of flexoelectricity regarding existing materialsand catalytic applications, thus showing the importance and relevance of this rapidly growing topic in literature. To conclude and summarize this section, we will introduce some perspectives and challenges in the field that might need to be addressed in the near future for a fully operational strain‐dependent catalytic system.

## 6 Perspectives and Challenges

Catalysis underpins countless industries yet faces critical efficiency, innovation, and clean energy production challenges. Flexoelectricity, a rapidly evolving field, offers a transformative perspective, reshaping how we design and utilize catalysts. Unlike traditional, static catalyst interactions, flexoelectricity introduces a dynamic interplay: mechanical strain on the catalyst material alters its electrical properties, influencing its interaction with molecules. This opens exciting possibilities that can be enhanced by combining photo‐active materials, especially in the following areas:

- Multifunctional reactors and energy harvesters: By tailoring the flexoelectric response, we can trigger distinct reaction mechanisms based on external stimuli. This enables synergistic reactions or low‐power energy generation from everyday activities like walking or running. Imagine generating electricity to power wearable devices simply through movement.
- Sustainable and cost‐effective solutions: Flexoelectric materials can be abundant and inexpensive, potentially replacing precious metal catalysts. This paves the way for cost‐effective clean energy technologies that are more accessible globally.
- Enhanced catalyst performance: Flexoelectricity's dynamic control can potentially prevent impurities from binding and deactivating the catalyst, leading to extended life and reliable performance.

### 6.1 Unlocking Flexoelectricity's Potential

Machine learning and advanced microfluidics/nanotechnology will be instrumental in unlocking the practical potential of flexoelectric catalysts. The aim for future investigations of flexoelectric materials and improvement should be focused on the two aspects. Starting with the theoretical one, the flexoelectric coefficient has yet to be physically explained thoroughly even in the simplest materials. The obtained theoretical value for known models is still too far away from the values observed experimentally. Going further to the experiments, the production and the invention of the new flexoelectric materials are still limited with a lack of knowledge about the mechanisms that are leading the flexoelectric effect to its severe enhancement, as mentioned in the photoflexoelectric effect and the flexophotocatalysis above.

Additionally, manipulating material geometry offers immense promise. Synergistically exploring flexoelectricity with piezoelectricity, ferroelectricity, and ferroelasticity (intrinsically linked phenomena) presents exciting possibilities. Moreover, artificial intelligence‐powered density functional theory (DFT) studies can be invaluable in deciphering this complex interplay, elucidating reaction mechanisms, and identifying promising, low‐cost, highly polarized flexoelectric materials. This comprehensive approach is the key to unlocking the full potential of flexoelectric catalysis for a sustainable future.

Perhaps more critical is the designation and implementation of several new topographies and geometries beyond suspended nanomaterials in fluids, allowing the recycling and extensive scalability of the strain‐driven catalyst. Additionally, the tuning geometrical aspects (such as length) on cantilevers/pillars, with given natural frequencies, might allow the exploitation of flexoelectric effect by naturally occurring vibrations, reducing the need for high‐power ultrasonic vibrations or other extra energy sources.

### 6.2 Beyond Clean Energy: A Broader Impact

Flexoelectric energy harvesters require a careful arrangement of material properties. Elegant nanostructuring is crucial, with the meticulous design of miniature architectures that, as mentioned before, could maximize the flexoelectric response. However, other qualities are essential: remarkable bending potential (like a flexible material that does not break, high flexural modulus, and superelastic properties), efficient charge mobility (ensuring smooth energy flow and heterojunctions), and durability to withstand everyday stresses. This would bring electrochemists and materials engineers to share ideas and, perhaps, devote efforts to generating ideal strain‐driven catalysts with exceptionally high yields. Furthermore, spreading flexoelectricity and flexocatalysis into other areas of science, especially in biomedicine, promises a broader impact on applicability and interdisciplinarity.

**Figure 22:**
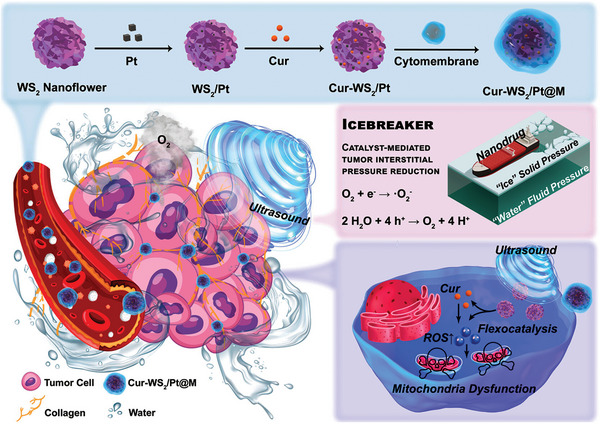
Schematic representation of Cur‐WS_2_/Pt@M, illustrating the enhancement of drug permeation and amplified antitumor efficacy within the tumor facilitated by curcumin and flexocatalysis. Image adapted with permission from the reference.^[^ ^262^ ^]^ Copyright 2024, American Chemical Society.

Quite recently, flexocatalytic applications in biomedical applications have been explored in the literature; the flexocatalysis technology coupled with curcumin presents a promising approach for addressing the challenges of solid tumor treatment in nanomedicine.^[^ ^262^ ^]^ By leveraging flexoelectricity induced through ultrasound, such a strategy reduces tumor interstitial fluid pressure (TIFP) and solid pressure caused by collagen overproduction (**Figure** **22**). Curcumin concurrently inhibits cancer‐associated fibroblasts (CAFs), further alleviating solid pressure within tumors. This combined approach enhanced the permeability of solid tumors to nanoscaled drugs, as demonstrated using a WS_2_/Pt Schottky heterojunction therapeutic platform. Overall, this highlights the significant potential of flexocatalysis and nanomedicine in improving cancer therapy outcomes, offering hope to patients, advancing oncological treatment, and opening the flexocatalytic field to biomedical applications.

Furthermore, in order to achieve maximum spread and outreach, any technology should meet the cost‐effectiveness limitation, which remains of paramount importance and limits the applications of many technologies; commercially viable harvesters are critical for widespread adoption and societal impact; in this aspect, the lack of symmetry restrictions opens a massive opportunity for many applications, preparing flexoelectric catalysts from inexpensive materials. However, several aspects and opportunities need to be explored, for instance, the role of hybrid composite interfaces on the overall performance of flexoelectric systems, many materials, and polymeric composites have shown promise in photo/catalytic applications, and strain effects might enhance their response by harvesting spurious vibrational energy or the turbulent flow. Additionally, mechanochemistry and sonochemistry provide a large bench for exploringstrain‐driven applications in selective processes beyond energy production, extending the applicability of this effect in many fields.

Finally, flexoelectricity's potential extends beyond clean energy, offering solutions for environmental protection and other industries. This emerging field holds immense promise for revolutionizing catalysis and shaping a more sustainable and efficient future for material science in general, while keeping the door open for many industries in which strain effects might not be as innocuous or negligible as traditionally seen.

## Conflict of Interest

The authors declare no conflict of interest.
